# Triblock copolymer micelle model of spherical paraspeckles

**DOI:** 10.3389/fmolb.2022.925058

**Published:** 2022-08-22

**Authors:** Tetsuya Yamamoto, Tomohiro Yamazaki, Tetsuro Hirose

**Affiliations:** ^1^ Institute for Chemical Reaction Design and Discovery, Hokkaido University, Sapporo, Japan; ^2^ PRESTO, Japan Science and Technology Agency (JST), 4-1-8 Honcho, Kawaguchi, Japan; ^3^ Graduate School of Frontier Biosciences, Osaka University, Suita, Japan; ^4^ Institute for Open and Transdisciplinary Research Initiatives, Osaka University, Suita, Japan

**Keywords:** paraspeckle, NEAT1_2, architectural RNA, micellization, transcription

## Abstract

Paraspeckles are nuclear bodies scaffolded by RNP complexes of NEAT1_2 RNA transcripts and multiple RNA-binding proteins. The assembly of paraspeckles is coupled with the transcription of NEAT1_2. Paraspeckles form the core-shell structure, where the two terminal regions of NEAT1_2 RNP complexes compose the shell of the paraspeckle and the middle regions of these complexes compose the core. We here construct a theoretical model of paraspeckles by taking into account the transcription of NEAT1_2 in an extension of the theory of block copolymer micelles. This theory predicts that the core-shell structure of a paraspeckle is assembled by the association of the middle region of NEAT1_2 RNP complexes due to the multivalent interactions between RBPs bound to these regions and by the relative affinity of the terminal regions of the complexes to the nucleoplasm. The latter affinity results in the effective repulsive interactions between terminal regions of the RNA complexes and limits the number of complexes composing the paraspeckle. In the wild type, the repulsive interaction between the middle and terminal block dominates the thermal fluctuation. However, the thermal fluctuation can be significant in a mutant, where a part of the terminal regions of NEAT1_2 is deleted, and distributes the shortened terminal regions randomly between the shell and the core, consistent with our recent experiments. With the upregulated transcription, the shortened terminal regions of NEAT1_2 in a deletion mutant is localized to the core to decrease the repulsive interaction between the terminal regions, while the structure does not change with the upregulation in the wild type. The robustness of the structure of paraspeckles in the wild type results from the polymeric nature of NEAT1_2 complexes.

## Introduction

A cell nucleus is not a uniform solution of DNA and proteins, but there are a number of nuclear bodies in the interchromatin space (Chujo et al., 2016; Nakagawa et al., 2018; Palikyaras and Papantonis 2019; Banani et al., 2017; Berry et al., 2015; Van Treeck and Parker 2018). Some nuclear bodies are scaffolded by RNAs that make ribonucleoprotein (RNP) complexes with RNA-binding proteins (RBPs). A class of RNAs that are essential in assembling nuclear bodies are called architectural RNAs (arcRNAs) (Chujo et al., 2016). Growing number of evidences suggest that nuclear bodies are condensates assembled by liquid-liquid phase separation (LLPS) because of the multivalent interactions between the intrinsically disordered regions of RBPs that are bound to arcRNAs (Chujo et al., 2016; Nakagawa et al., 2018; Palikyaras and Papantonis 2019; Banani et al., 2017; Berry et al., 2015; Van Treeck and Parker 2018). Condensates produced by LLPS are thought to act as reaction crucibles of specific biochemical reactions, molecular sponges that sequester specific proteins and RNAs from the nucleoplasm, and hubs to organize the 3D structure of genome (Shin and Brangwynne 2017). It is of interest to study the assembly mechanism of nuclear bodies due to the possible relationship with their physiological functions.

Paraspeckles are nuclear bodies that are scaffolded by NEAT1_2 arcRNA (Sunwoo et al., 2008; Clemson et al., 2009; Sasaki et al., 2009). Paraspeckles act as molecular sponges that sequester some types of RNA transcripts and proteins (Chen and Carmichael 2009; Hirose et al., 2014; Imamura et al., 2014; Hu et al., 2015; West et al., 2016; Nakagawa et al., 2018; Wang et al., 2018; Yap et al., 2022) and interact with chromatin regions, enriched in active promoters and enhancers (West et al., 2014; Li et al., 2017; Sridhar et al., 2017; Fang et al., 2019; Bonetti et al., 2020; Cai et al., 2020). *De novo* assembled paraspeckles are often observed at the proximity to the transcription site of NEAT1_2 and are disassembled when the transcription of arcRNA is suppressed (Clemson et al., 2009; Sasaki et al., 2009; Mao et al., 2011). The number of paraspeckles increases when transcription of NEAT1_2 is upregulated (Clemson et al., 2009; Hirose et al., 2014). These results imply that the assembly of paraspeckles is coupled with the transcription of NEAT1_2. RNA-binding proteins can be bound to nascent NEAT1_2 transcripts, which are still connected to the transcription site *via* RNA polymerase II (Pol II). The array of nascent NEAT1_2 RNAs produced during a transcription burst probably act as a nucleation site of paraspeckles (Chujo and Hirose 2017; Yamazaki et al., 2020).

We have recently extended the Flory-Huggins theory, which is the standard theory of phase separation of polymers in a solution (Doi 1996), to predict the phase separation driven by the production of arcRNAs due to transcription (Yamamoto et al., 2020). The condensates assembled by this mechanism are disordered liquids of complexes of arcRNAs and RBPs. However, paraspeckles are not condensates of disordered liquid, but form a characteristic core-shell structure (Souquere et al., 2010; West et al., 2016): the two terminal regions and the middle region of NEAT1_2 form the shell and the core of paraspeckles, respectively. The structure of paraspeckles is analogous to micelles of ABC triblock copolymers in a selective solvent, where two polymer chains (A and C blocks) composed of hydrophilic units are chemically bonded to the two ends of a chain (B block) composed of hydrophobic units (Monzen et al., 2000; Mai and Eisenberg 2012; Moughton et al., 2012). The ordered structure and the transcription driven formation of paraspeckles are two features that distinguish paraspeckles from condensates assembled by the classical phase separation, such as LLPS, in the thermodynamic equilibrium.

**FIGURE 1:**
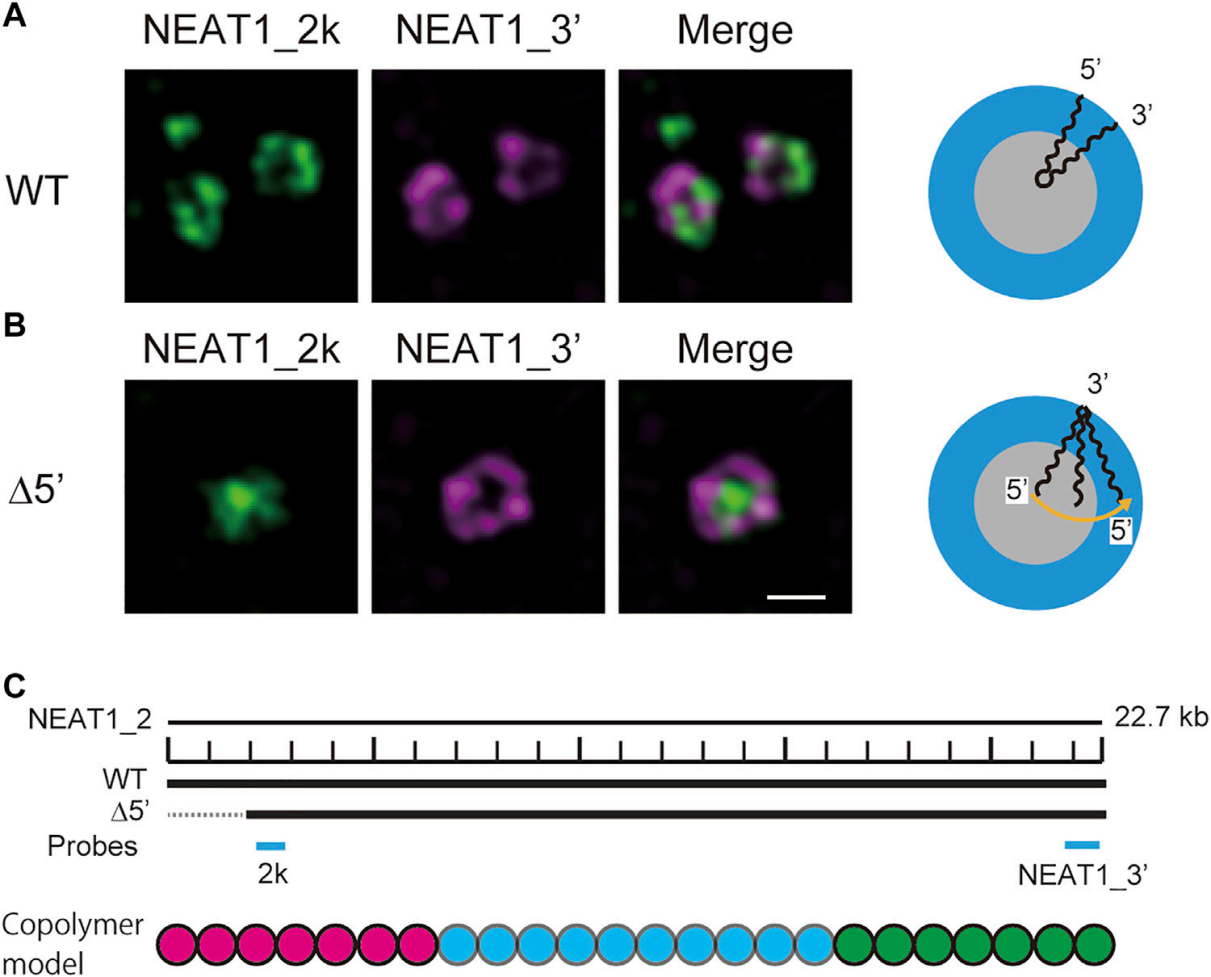
Super-resolution microscopic images of paraspeckles in HAP1 NEAT1 wild type **(A)** and D5′ mutant cells lacking their NEAT1 0–1.8 kb regions **(B)** detected by NEAT1_2k FISH probes against 5′ terminal region of NEAT1 (green) and NEAT1_3′ FISH probes (magenta) in the presence of MG132 (5 mM for 6 h). Scale bar, 500 nm. **(C)** The schematics of WT NEAT1_2 and mutants with deletions in the 5′ terminal regions. The positions of NEAT1 probes (NEAT1_2k and NEAT1_3′) are shown by the blue bars. See Yamazaki et al. 2021 for experimental details. Copolymer model is schematic and the borders of A, B, and C blocks remain to be experimentally characterized.

In our recent experiments, we have constructed mutant NEAT1_2 cell lines, in which the terminal regions of NEAT1_2 were partly deleted by CRISPR/Cas9 and have observed paraspeckles in such cell lines with the super-resolution optical microscope and the electron microscope (Yamazaki et al., 2018, 2021). Our experiments have shown that the terminal regions of NEAT1_2 RNAs were localized in the shells of paraspeckles in the wild type (WT) (Figure 1A), whereas the terminal regions were distributed both to the core and the shell in the mutant cells (Figure 1B) (the schematics of NEAT1_2 for each case is shown in Figure 1C). Motivated by this result, we here construct a model of paraspeckles by taking into account the transcription dynamics of NEAT1_2 in an extension of the theory of micelles of ABC triblock copolymers. The A and C blocks correspond to the terminal regions of NEAT1_2 RNP complexes and the B block corresponds to their middle region. The B blocks are associative due to the multivalent interactions between RBPs, such as NONO and FUS, that specifically bind to this B block region due to its sequence (Yamazaki et al., 2018).

In many cases, paraspeckles are approximately spherical, whereas cylindrical paraspeckles are also observed, for example, when NEAT1_2 transcription is upregulated (Hirose et al., 2014). The analysis of cylindrical paraspeckles greatly complicates the theory, while the essence of the biophysical mechanism of the assembly of paraspeckles is already in the theory of spherical paraspeckles. Not to hide the essence behind the complexity of analysis, in this paper, we limit our discussion to spherical paraspeckles.

Our theory predicts the distribution of terminal blocks (A blocks) and the size of paraspeckles in wild type and mutant with deleted terminal blocks and also the effect of the upregulation of NEAT1_2 transcription. Our prediction is consistent with our recent experiments (Yamazaki et al., 2018, 2021), implying that the assembly of paraspeckles can be understood as micellization. Our theory provides biophysical insight into the assembly of WT and mutant paraspeckles. The sequences of arcRNAs determine the arrangements of RBPs along these arcRNAs and thus play a role in the blueprints of nuclear bodies, while their assembly is fine-tuned by the transcription dynamics of arcRNAs. Our theory can be extended to understand the mechanism of the assembly of other nuclear bodies once the arrangements of RBPs along arcRNAs are determined by experiments.

## Materials and methods

### Model

**FIGURE 2:**
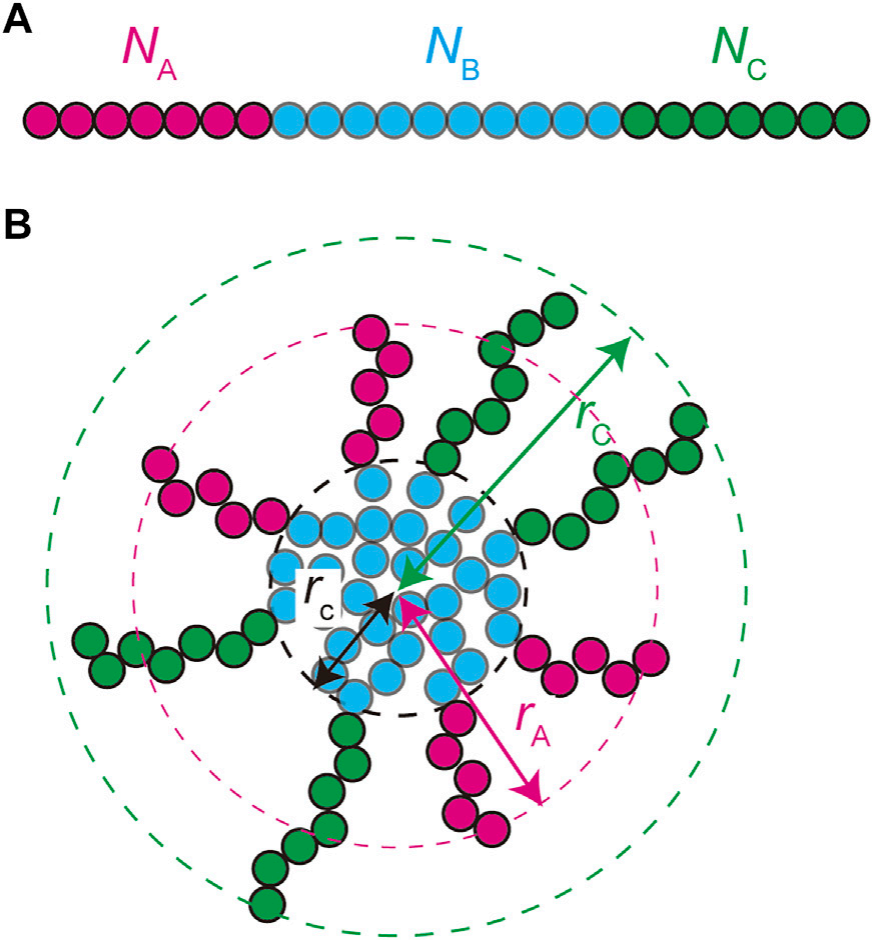
A spherical paraspeckle is modeled as a micelle of ABC block copolymers. The A, B, and C blocks are composed of $N_{\text{A}}$ , $N_{\text{B}}$ , and $N_{\text{C}}$ units, respectively **(A)**. Each paraspeckle is composed of *n* copolymers. The B blocks of the copolymers are packed in the core of the paraspeckle and the C blocks are localized in the shell **(B)**. A fraction α of the A blocks is localized at the shell and the other fraction is in the core. The A and C blocks are located in distinct domains in the shell. The size of the paraspeckle is characterized by the radius $r_{\text{c}}$ of the core, the distance $r_{\text{A}}$ between the top of an A domain and the center of the paraspeckle, and the distance $r_{\text{C}}$ between the top of a C domain and the center of the paraspeckle.

We treat the NEAT1_2 RNP complexes as ABC triblock copolymers (Figure 2A). Different RBPs are bound to A, B, and C blocks and this makes the magnitudes of interactions between units in the same blocks different from the magnitudes of the interactions between different blocks. Treating a complex as one polymer, instead of treating arcRNA and RBPs separately, is effective for the case in which the binding affinity of RNA-binding proteins to these blocks is relatively large (Yamamoto et al., 2020). The A, B, and C blocks are composed of $N_{\text{A}}$ , $N_{\text{B}}$ , and $N_{\text{C}}$ (Kuhn) units, respectively, (Figure 2A). The core of a paraspeckle is assembled by the association of the B blocks because of the attractive interactions between the RBPs bound to these blocks (Figure 2B). The A and C blocks form the shell because the RBPs bound to these terminal blocks have affinity to the solution (nucleoplasm), rather than the RBPs bound to B blocks. The analysis of the cylindrical paraspeckles greatly complicates the theory because of the massive form of the free energy, an extra geometrical parameter that should be determined by minimizing the free energy, and the lack of steady state. In this paper, we focus on spherical paraspeckles.

In the thermodynamic equilibrium, the most stable state is the one at the minimum of the free energy of the system. The free energy of a micelle of block copolymers is composed of 1) the stretching free energy of the blocks in the core, 2) the surface free energy at the interface between the core and the shell, and 3) the free energy of the blocks in the shell (Halperin and Alexander 1989; Semenov et al., 1995; Zhulina et al., 2005). The growth of polymer micelles decreases the surface free energy and increases the stretching free energy of blocks in the core and the free energy due to the excluded volume interactions between blocks in the shell (Halperin and Alexander 1989; Semenov et al., 1995; Zhulina et al., 2005). The stable size of spherical micelles is determined by the balance of these free energy contributions. Most theories predict the distribution of the size of polymer micelles that are assembled uniformly in a block copolymer solution at the thermodynamic equilibrium (Annianson and Wall 1974; Safran 2003; Hadgiivanova et al., 2011; Mysona et al., 2019). We extend the theory of polymer micelles by taking into account the fact that paraspeckles are assembled locally at the transcription site of NEAT1_2 and their assembly is coupled with the transcription of NEAT1_2.

### Free energy

**FIGURE 3:**
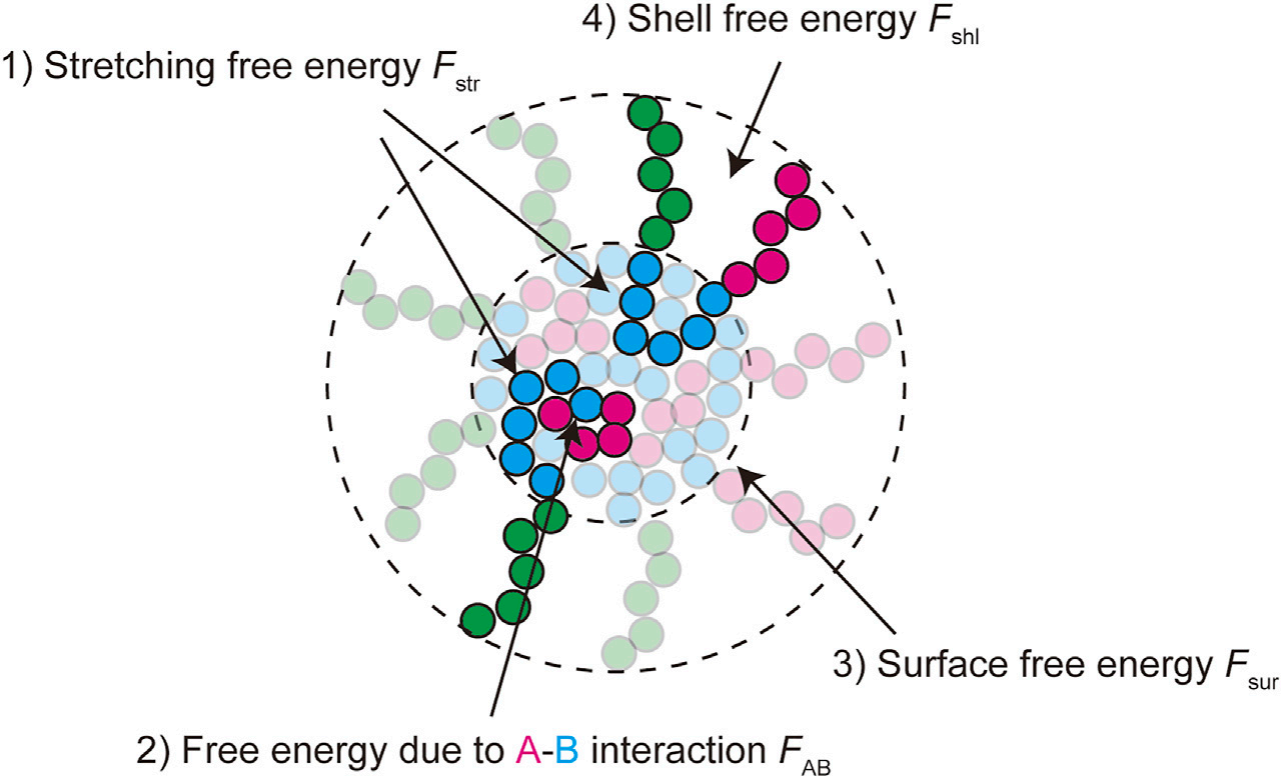
The free energy of a paraspeckle is composed of five terms: 1) the stretching free energy of blocks in the core $F_{\text{str}}$ , 2) the free energy due to the repulsive interactions between A and B units in the core $F_{\text{AB}}\;\left(\approx \chi_{\text{AB}}N_{\text{A}}n\right)$ , 3) the surface free energy $F_{\text{sur}}$ $\left(\approx N_{B}^{-1/3}n^{5/3}\right)$ , 4) the free energy of the shell $F_{\text{shl}}$ $\left(\approx N_{A}N_{B}^{-5/9}n^{23/18}\right)$ , and 5) the mixing free energy $F_{\text{mix}}$ . The free energy contributions $F_{\text{AB}}$ and $F_{\text{sur}}$ represent the (free) energetic penalty because B units at the vicinity of A units in the core and at the surface have a lesser number of partners of multivalent interactions.

The free energy quantifies the stability of the system at the thermodynamic equilibrium. We here derive the free energy of a paraspeckle by taking into account the fact that the terminal blocks of NEAT1_2 are distributed both to the shell and the core in an extension of the free energy of a polymer micelle (Halperin and Alexander 1989; Semenov et al., 1995; Zhulina et al., 2005). The free energy of a paraspeckle composed of n triblock copolymers has the form

$$\begin{array}{c} F_{n}=F_{\text{cor}}+F_{\text{sur}}+F_{\text{shl}}+F_{\text{mix}} \end{array},$$ (1)

where $F_{\text{cor}}$ is the free energy of the core, $F_{\text{sur}}$ is the surface free energy at the interface between the core and the shell, $F_{\text{shl}}$ is the free energy of the shell, and $F_{\text{mix}}$ is the mixing free energy (Figure 3).

The free energy of the core has the form

$$\begin{array}{c} \frac{F_{\text{cor}}}{k_{\text{B}}T}=\frac{3}{2}\lambda_{\text{s}}\frac{r_{\text{c}}^{5}}{N_{\text{B}}^{2}b^{5}}4\alpha +\frac{3}{2}\lambda_{\text{s}}\frac{r_{\text{c}}^{5}}{\left(N_{\text{A}}+N_{\text{B}}\right)b^{5}}\left(1-\alpha \right)+\chi_{AB}\phi_{A}\phi_{B}\frac{V_{\text{c}}}{b^{3}} \end{array}.$$ (2)

The first and second terms are the stretching free energy of the B blocks in the core and are derived in the spirit of Semenov 1985, see Supplementary Section S1. The third term is the free energy due to the interactions between the A and B units in the core. For cases in which the number $N_{\text{A}}$ of the A blocks is relatively large, these blocks may aggregate to minimize the repulsive interactions between A and B units. However, to keep the simplicity of the theory, we derived the third term of Eqn. 2 by assuming that the fraction 1−α of the A blocks are distributed uniformly in the core, independent of the number $N_{\text{A}}$ of units in A blocks. With the assumption with which the core is packed with A and B blocks, the volume $V_{\text{c}}$ has the form

$$\begin{array}{c} V_{\text{c}}=\frac{4\pi }{3}r_{\text{c}}^{3}=nb^{3}\left(N_{\text{B}}+\left(1-\alpha \right)N_{\text{A}}\right) \end{array}$$ (3)

where $r_{\text{c}}$ is the radius of the core. $\lambda_{s}\;\left(=\pi^{3}/30\right)$ is the geometrical factor (Semenov 1985). The interaction parameter $\chi_{\text{AB}}$ accounts for the excluded volume interactions between the A blocks and the B blocks. $\phi_{\text{A}}(=b^{3}\left(1-\alpha \right)N_{\text{A}}n/V_{\text{c}})$ and $\phi_{\text{A}}\left(=b^{3}N_{\text{B}}n/V_{\text{c}}\right)$ are the volume fractions of the A blocks and the B blocks in the core, respectively. The core is occupied by the A and B blocks, $\phi_{\text{A}}+\phi_{\text{B}}=1$ .

The surface free energy has the form

$$\begin{array}{c} F_{\text{sur}}=4\pi \chi_{B}\frac{r_{c}^{2}}{b^{2}} \end{array}.$$ (4)

The interaction parameter $\chi_{\text{B}}$ accounts for the (free) energetic penalty due to the fact that the B units at the surface have less number of interacting partners than in the interior of the core. Without changing the physics, we treat cases in which the A and C units are dilute both in the core and the shell. In such cases, the interactions between the B units at the core surface and the A units in the shell as well as the interactions between the A units at the core surface and the solution are both negligible.

Theories of block copolymer micelles treat blocks in the shell as a polymer brush on a curved surface (Halperin and Alexander 1989; Semenov et al., 1995; Zhulina et al., 2005). There are elaborate approaches to treat polymer brushes on planer (Netz and Schick 1998) and curved surfaces (Zhulina et al., 2006), however, for simplicity, we here use the scaling theory of polymer brush (Alexander 1977; de Gennes 1980). With this approach, the free energy of the shell has the form

$$\begin{array}{c} F_{\text{shl}}=F_{\text{A}}+F_{\text{C}} \end{array}$$ (5)

with

$$\begin{array}{c} \frac{F_{\text{A}}}{k_{\text{B}}T}=\frac{3}{5}\frac{{\left(n\alpha \right)}^{\frac{3}{2}}}{{\left(4\pi f_{\text{A}}\right)}^{\frac{1}{2}}}C_{\text{s}}\log\left(1+\frac{5}{3}\frac{h_{\text{A}}}{r_{\text{c}}}\right) \end{array}$$ (6)

$$\begin{array}{c} \frac{F_{\text{A}}}{k_{\text{B}}T}=\frac{3}{5}\frac{n^{\frac{3}{2}}}{{(4\pi \left(1-f_{\text{A}}\right))}^{\frac{1}{2}}}C_{\text{s}}\log\left(1+\frac{5}{3}\frac{h_{\text{C}}}{r_{\text{c}}}\right) \end{array}.$$ (7)

Eqn. 6 includes the stretching free energy of the A blocks in the shell and the free energy due to the excluded volume interactions between A units in the shell. Eqn. 7 is the corresponding free energy for the C blocks. We neglected the excluded volume interaction between the A and C units because the 3′ and 5’ terminal regions are segregated in separate domains in the shell of wild type paraspeckles (West et al., 2016). Eqs. 6, 7 are derived by using so-called Daoud-Cotton scaling theory (Daoud and Cotton 1982), assuming that the solution is a good solvent to both A and C blocks, following the usual treatment of block copolymer micelles (Halperin and Alexander 1989; Semenov et al., 1995; Zhulina et al., 2005), see also in Supplementary Section S2.1 $f_{\text{A}}$ is the fraction of the core surface occupied by A blocks and has the form

$$\begin{array}{c} f_{A}=\frac{\alpha }{1+\alpha } \end{array}.$$ (8)

Eqn. 8 is derived by using the fact that the osmotic pressure in the domains of A blocks is equal to the osmotic pressure in the domains of C blocks, see Supplementary Section S2.1. $h_{\text{A}}$ and $h_{\text{C}}$ are the heights of A and C blocks in the limit of planer brush, $r_{\text{c}}\to \infty$ , respectively. These heights have the forms (Alexander 1977; de Gennes 1980)

$$\begin{array}{c} h_{\text{A}}=N_{A}b{\left(v_{\text{A}}\frac{n\alpha }{4\pi f_{\text{A}}r_{\text{c}}^{2}b}\right)}^{\frac{1}{3}} \end{array}$$ (9)

$$\begin{array}{c} h_{\text{C}}=N_{\text{C}}b{\left(v_{\text{C}}\frac{n}{4\pi \left(1-f_{\text{A}}\right)r_{\text{c}}^{2}b}\right)}^{\frac{1}{3}} \end{array}.$$ (10)

$v_{\text{A}}$ and $v_{\text{C}}$ are the excluded volumes that account for the excluded volume interactions between the A units and those between the C units, respectively. $C_{\text{s}}$ is a numerical constant of order unity and is determined as $C_{\text{s}}≃1.38$ by curvefitting the experiments on the micelles of polystylene-polyisoprene copolymers with the scaling theory (Zhulina et al., 2005).

The mixing free energy represents the entropic contribution that distributes the A blocks randomly to the shell and the core and has the form

$$\begin{array}{c} \frac{F_{\text{mix}}}{k_{\text{B}}T}=n\left(\alpha \log\alpha +\left(1-\alpha \right)\log\left(1-\alpha \right)\right) \end{array}.$$ (11)

The free energy $F_{n}\left(\alpha \right)$ is a function of the number *n* of triblock copolymers comprising the paraspeckle and the fraction α of A blocks in the shell. We determine the fraction α by the minimization of the free energy $F_{n}\left(\alpha \right)$ . This corresponds to cases in which the time scale of the redistribution of A blocks is smaller than the time scale of the production of transcripts.

### Association and dissociation dynamics of NEAT1_2

Most theories of polymer micelles predict the most stable size and the distribution of the size of polymer micelles that are assembled uniformly in a block copolymer solution at the thermodynamic equilibrium (Annianson and Wall 1974; Safran 2003; Hadgiivanova et al., 2011; Mysona et al., 2019). In contrast, the assembly of paraspeckles is coupled with the transcription of NEAT1_2 (Mao et al., 2011). RBPs can bind to nascent NEAT1_2 transcripts, which are still connected to the transcription site via Pol II, and an array of complexes of nascent NEAT1_2 transcripts and RBPs during a transcription burst act as a nucleation site of paraspeckles. Once a paraspeckle is nucleated, nascent NEAT1_2 transcripts are added to the paraspeckle one by one as nascent transcripts are produced by Pol II. This situation may be somewhat analogous to the assembly of micelles due to the association of side chains that are attached to a main chain, except for the fact that these side chains are not produced and released dynamically. The side chains assemble micelles without translational entropy cost, in contrast to the micellization of polymer chains freely diffusing in the solution (Borisov and Halperin 1995). We thus derive the time evolution equation of a paraspeckle at the transcription site by taking into account the facts that 1) nascent NEAT1_2 transcripts are associated with the paraspeckle without the translational entropy cost and 2) paraspeckles are assembled locally at the transcription site. Because of these features, paraspeckles are different from block copolymer micelles assembled in the thermodynamic equilibrium (Annianson and Wall 1974; Safran 2003; Hadgiivanova et al., 2011; Mysona et al., 2019).

We treat the probability $q_{n}\left(t\right)$ that the paraspeckle assembled at the transcription site of NEAT1_2 is composed of n transcripts at time t . The time evolution of the probability $q_{n}\left(t\right)$ has the form

$$\begin{array}{c} \frac{d}{dt}q_{1}\left(t\right)=-J_{1}\left(t\right) \end{array}$$ (12)

$$\begin{array}{c} \frac{d}{dt}q_{n}\left(t\right)=-J_{n}\left(t\right)+J_{n-1}\left(t\right) \end{array}.$$ (13)

The flux $J_{n}\left(t\right)$ has the form

$$\begin{array}{c} J_{n}\left(t\right)=k_{0}\phi_{\text{p}}q_{n}\left(t\right)-k_{0}e^{-\left(F_{n}+F_{1}-F_{n+1}\right)/\left(k_{B}T\right)}\left(n+1\right)q_{n+1}\left(t\right)+k_{\text{tx}}q_{n}\left(t\right) \end{array}.$$ (14)

The first term of Eqn. 14 is the rate with which transcripts diffusing in the solution are spontaneously associated with the paraspeckle. The second term is the rate with which a transcript is spontaneously dissociated from the paraspeckle. The third term is the rate with which a nascent transcript during production is added to the paraspeckle through transcription. $k_{0}$ is the rate constant that accounts for the association of transcripts diffusing in the solution with the paraspeckle. $\phi_{\text{p}}$ is the volume fraction of transcripts that are not associated to paraspeckles. $F_{n}$ is the free energy which is already minimized with respect to the fraction α , see Eqn. 1. $k_{\text{tx}}$ is the rate with which a nascent transcript is added to the paraspeckle. The form of the third term of Eqn. 14 represents the fact that nascent transcripts can be associated with the paraspeckle without the translational entropy cost. We note that the probability $q_{n}\left(t\right)$ is the local quantity of the paraspeckle assembled at the transcription site, in contrast to the usual treatment of micelles that predict the global distribution function of the size of micelles in a solution (Annianson and Wall 1974; Safran 2003; Hadgiivanova et al., 2011; Mysona et al., 2019).

For cases in which the transcription is suppressed, $k_{\text{tx}}\to 0$ , the probability $q_{n}^{\text{eq}}$ in the equilibrium has the form

$$\begin{array}{c} q_{n}^{\text{eq}}\propto \frac{\phi_{\text{p}}^{n-1}}{\left(n-1\right)!}{\text{e}}^{-\frac{F_{n}-nF_{1}}{k_{\text{B}}T}} \end{array}.$$ (15)

Substituting Eqn. 15 into Eqn. 14 leads to $J_{n}=0$ for $k_{\text{tx}}\to 0$ that ensure the detailed balance at the thermodynamic equilibrium.

In the steady state, $\frac{dq\left(t\right)}{dt}=0$ , the solution of Eqs. 12, 13 has the form

$$\begin{array}{c} q_{n}^{\text{st}}=\frac{1}{Z_{\text{st}}}e^{-\frac{{ℱ}_{n}^{\text{st}}}{k_{\text{B}}T}} \end{array}.$$ (16)

The effective free energy ${ℱ}_{n}^{\text{st}}$ has the form

$$\begin{array}{c} {ℱ}_{n}^{\text{st}}=F_{n}-nF_{1}-k_{B}T\left(n-1\right)\log\left(\phi_{\text{p}}+\zeta \right)+k_{\text{B}}T\log\left(n!\right) \end{array}$$ (17)

where $\zeta \;\left(=k_{\text{tx}}/k_{0}\right)$ is the ratio of the association rates. $Z_{\text{st}}$ is the effective partition function

$$\begin{array}{c} Z_{st}=\overset{\infty }{\underset{n=1}{\sum }}e^{-{ℱ}_{n}^{\text{st}}/\left(k_{\text{B}}T\right)} \end{array}.$$ (18)

The number of transcripts composing a paraspeckle with the maximum probability in the steady state is derived by minimizing the effective free energy ${ℱ}_{n}^{\text{st}}$ with respect to n . In this paper, we treat cases in which NEAT1_2 transcripts are exclusively localized in paraspeckles, $\phi_{p}\to 0$ (Sasaki et al., 2009; Yamazaki et al., 2021).

## Results

### Transcription dynamics regulates the structure of paraspeckles

**FIGURE 4:**
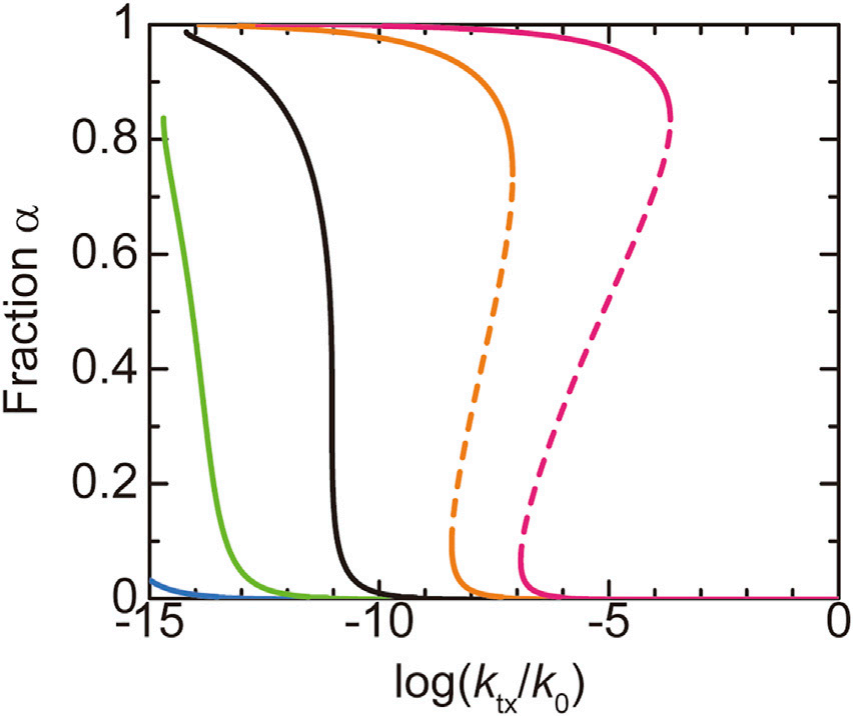
The fraction α of A blocks in the shell of a paraspeckle is shown as a function of the (natural) logarithm of the transcription rate $k_{\text{tx}}$ for $N_{\text{A}}$ = 1.0 (cyan), 3.0 (light green), 5.2483 (black), 8.0 (orange), and 10.0 (magenta). The values of parameters used for the calculations are $N_{\text{B}}$ = 40.0, $N_{\text{C}}$ = 15.0, $\chi_{\text{B}}$ = 0.5, $\chi_{\text{AB}}$ = 1.0, $v_{\text{A}}/b^{3}=v_{\text{C}}/b^{3}$ = 1.0, and $C_{\text{s}}$ = 1.5.

**FIGURE 5:**
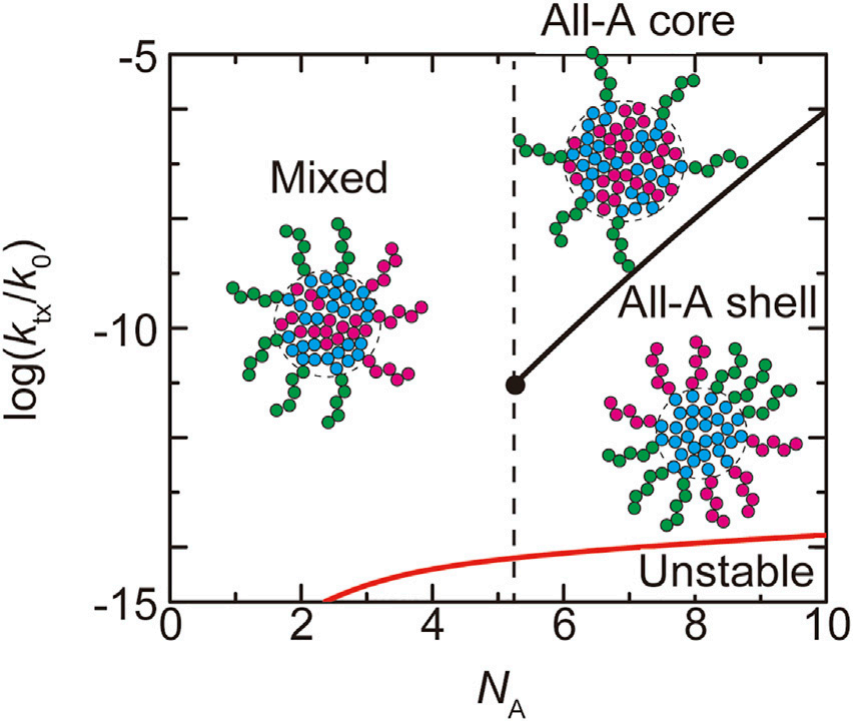
The phase diagram of paraspeckles is shown for the (natural) logarithm of production rate $k_{\text{tx}}$ of transcripts and the number $N_{\text{A}}$ of units in the A blocks. Paraspeckles are not stable in the region delineated by the red line. The values of parameters used for the calculations are $N_{\text{B}}$ = 40.0, $N_{\text{C}}$ = 15.0, $\chi_{\text{B}}$ = 0.5, $\chi_{\text{AB}}$ = 1.0. $v_{A}/b^{3}=v_{C}/b^{3}$ = 1.0, and $C_{\text{s}}$ = 1.5. The vertical broken line indicates the critical number $N_{\text{Ac}}$ of units in the A blocks.

We first discuss the dependence of the fraction α of A blocks in the shell on the production rate $k_{\text{tx}}$ of NEAT1_2 transcripts. The number n of transcripts in a paraspeckle is derived as a function of $k_{\text{tx}}$ by minimizing the effective free energy ${ℱ}_{n}^{\text{st}}$ , see Eqn. 17, and the fraction α  of A blocks in the shell is derived by minimizing the free energy $F_{n}$ for this number n , see Eqn. 1. When the number $N_{\text{A}}$ of units in the A blocks is smaller than a critical value $N_{\text{Ac}}$ , the fraction α decreases continuously with increasing the production rate $k_{\text{tx}}$ (the cyan and light green lines in Figure 4). When the number $N_{\text{A}}$ of units in the A blocks is larger than the critical value $N_{\text{Ac}}$ , the paraspeckles are the ‘all-A shell’ state, in which all the A blocks are in the shell, α≃1 , for low transcription rate and the ‘all-A core’ state, in which all the A blocks are in the core, α≃0 , for high transcription rate (the orange and magenta lines in Figure 4). There is a discontinuous transition between the all-A shell and all-A core states at a threshold value of production rate. This result is summarized in the phase diagram (Figure 5). The ‘mixed’ state for $N_{\text{A}}<N_{\text{Ac}}$ , where A blocks are distributed between the core and the shell, results from the thermal fluctuation, which is quantified by the mixing free energy $F_{\text{mix}}$ . For $N_{\text{A}}>N_{\text{Ac}}$ , the interaction free energy, $F_{\text{shl}}$ and $F_{\text{AB}}$ , where both scale proportional to $N_{\text{A}}$ , dominates the mixing free energy $F_{\text{mix}}$ , which is independent of $N_{\text{A}}$ and thus only the all-A shell and all-A core states are possible (see a quantitative argument below). It is analogous to the Flory-Huggins theory that predicts that the interaction free energy dominates the mixing free energy in polymer systems (Doi 1996). The fraction α of A blocks in the shell is sensitive to the upregulation of the transcription of NEAT1_2 transcripts when $N_{\text{A}}<N_{\text{Ac}}$ , whereas the fraction α does not change significantly by the moderate upregulation of the transcription when $N_{\text{A}}>N_{\text{Ac}}$ (Figure 4). It is because for $N_{\text{A}}>N_{\text{Ac}}$ , the interaction free energy dominates the mixing free energy and thus the changes of the fraction α is suppressed.

In the WT, most of the terminal regions of NEAT1_2 are localized at the shell (Souquere et al., 2010; West et al., 2016; Yamazaki et al., 2018, 2021). This implies that paraspeckles in the WT are in the ‘all-A shell’ state, which happens for $N_{\text{A}}>N_{\text{Ac}}$ and relatively low transcription rate. In deletion mutants, a fraction of the terminal blocks, which were partially deleted, is localized at the core, implying that paraspeckles in the deletion mutants correspond to the case of $N_{\text{A}}<N_{\text{Ac}}$ . Our recent experiments have shown that in deletion mutants, the fraction α of the terminal regions, which were partially deleted, localized at the shell decreases by the upregulation of the transcription of NEAT1_2, whereas in the WT, the fraction does not change with the upregulation of transcription (Yamazaki et al., 2021). These predictions are consistent with the prediction of our theory. However, we note that in the WT, the fraction of cylindrical paraspeckles increases by the upregulation (Hirose et al., 2014) and the morphological transition to cylinder may also be involved in the insensitivity.

Our theory predicts the biophysical mechanism of the assembly of paraspeckles. The fact that all the terminal blocks are localized at the shell in the WT paraspeckles results from the strong repulsive interaction between A and B blocks, where its influence to the structures of paraspeckles is quantified by the free energy $F_{\text{AB}}$ $\left(\approx \chi_{\text{AB}}N_{\text{A}}n\right)$ . Indeed, the free energy $F_{\text{shl}}$ $\left(\approx N_{A}N_{B}^{-5/9}n^{23/18}\right)\;$ of A and C blocks in the shell and the free energy $F_{\text{str}}\;\left(\approx N_{B}^{-1/3}n^{5/3}\right)$ due to the stretching of B blocks in the core both decrease as the fraction α of A blocks in the shell decreases. However, the free energy $F_{\text{AB}}$ dominates probably because the magnitude $\chi_{\text{AB}}$ of the interaction between A and B blocks and the number $N_{\text{B}}$ of units in each B block is large enough in WT NEAT1_2. The influence of the thermal fluctuation that randomly distributes A blocks between the core and the shell to the structure is quantified by the mixing free energy $F_{\text{mix}}$ and it is independent of the number $N_{\text{A}}$ of units in A blocks. In contrast, the free energy $F_{\text{AB}}$ due to the interactions between A and B blocks in the core increases as the number $N_{\text{A}}$ of units in each A block increases. For $N_{\text{A}}>N_{\text{Ac}}$ , the interaction free energy $F_{\text{AB}}$ dominates the mixing free energy $F_{\text{mix}}$ and thus the ‘all-A shell’ state becomes stable. In contrast, for $N_{\text{A}}<N_{\text{Ac}}$ , the mixing free energy is still significant and thus A blocks are distributed between the shell and the core. This explains the difference of the structures of paraspeckles between WT and deletion mutants (Figure 1 and Yamazaki et al., 2018, 2021).

The number n of transcripts in the paraspeckle increases with increasing the transcription rate. For a relatively small transcription rate, all-A shell state becomes stable because the free energy $F_{\text{AB}}$ due to the repulsive interactions between A and B blocks in the core (see Eqn. 2 for $N_{B}>N_{\text{A}}$ ) dominates the free energy $F_{\text{shl}}\;$ due to the excluded volume interactions between A units in the shell and the stretching free energy $F_{\text{str}}$ of blocks in the core (see Eqn. 2 for $N_{\text{B}}>N_{\text{A}}$ ). The free energy $F_{\text{shl}}$ of the blocks in the shell and the stretching free energy $F_{\text{str}}$ of blocks in the core increase faster than the free energy $F_{\text{AB}}$ due to the repulsive interactions between A and B blocks in the core as the number n of transcripts in the paraspeckle increases. This results in the decrease of the fraction α of the A blocks in the shell with increasing the transcription rate (see the Supplementary Discussion for the relative significance of the free energy contributions $F_{\text{shl}}$ and $F_{\text{str}}$ ). For very small production rate, stable paraspeckles are not assembled, see the region delineated by the red line in Figure 5.

### Repulsive interactions of terminal blocks and entropic elasticity of middle blocks limit the number of transcripts in paraspeckles

**FIGURE 6:**
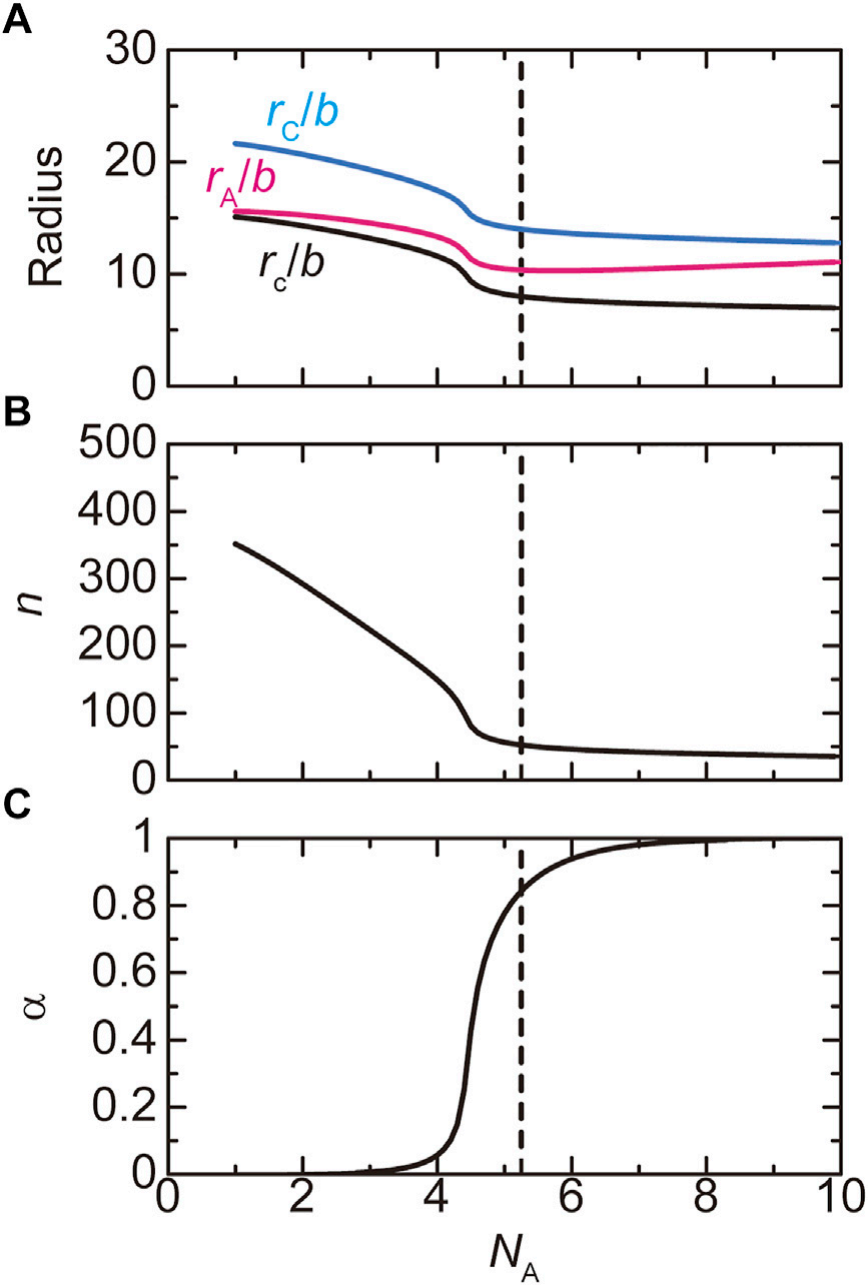
The radius **(A)**, the number n of copolymers **(B)**, and the fraction α of A blocks in the shell **(C)** of paraspeckles are shown as functions of the number *N*_A_ of units of the A blocks. In a, we showed the radius $r_{\text{c}}$ of the core of a paraspeckle (black), the distance $r_{\text{A}}$ between the top of the A blocks and the center of the paraspeckle (magenta), and the distance $r_{\text{C}}$ between the top of C blocks and the center of the paraspeckle (cyan). The (natural) logarithm $\log\left(k_{\text{tx}}/k_{0}\right)$ of the production rate of copolymers is fixed to -12.0. The values of parameters used for the calculations are $N_{\text{B}}$ = 40.0, $N_{\text{C}}$ = 15.0, $\chi_{\text{B}}$ = 0.5, $\chi_{\text{AB}}$ = 1.0, $v_{A}/b^{3}=v_{C}/b^{3}$ = 1.0, and $C_{\text{s}}$ = 1.5. The vertical broken line indicates the critical number $N_{\text{Ac}}$ of units in A blocks.

The size of paraspeckles in the WT and deletion mutants is experimentally accessible. The radius $r_{\text{c}}$ of the core is derived by using Eqn. 3. The radii, $r_{\text{A}}$ and $r_{\text{C}}$ , are derived by using the forms

$$\begin{array}{c} r_{\text{A}}=r_{\text{c}}{\left(1+\frac{5}{3}\frac{h_{\text{A}}}{r_{\text{c}}}\right)}^{\frac{3}{5}} \end{array}$$ (19)

$$\begin{array}{c} r_{\text{C}}=r_{\text{c}}{\left(1+\frac{5}{3}\frac{h_{\text{C}}}{r_{\text{c}}}\right)}^{\frac{3}{5}} \end{array},$$ (20)

where the heights, $h_{\text{A}}$ and $h_{\text{C}}$ , are given in Eqs. 9, 10. The derivation of Eqs. 19, 20 are shown in Supplementary Section S2.1. These radii are functions of the number n of transcripts in a paraspeckle and the fraction α of blocks in the shell, where the latter parameters are derived similarly to Figure 4. At a first glance, one may think that the radius of the paraspeckle decreases as the number $N_{\text{A}}$ of units in A blocks decreases. However, our theory predicts that the radius of paraspeckles (defined by the distance between the top of the A or C blocks and the center of the paraspeckle) increases with decreasing the number $N_{\text{A}}$ of units in A blocks, see Figure 6A. It is because the number n of transcripts in the paraspeckle increases with decreasing the number $N_{\text{A}}$ of units in A blocks, see Figure 6B. This prediction is consistent with our recent experiments (Yamazaki et al., 2021).

The number of transcripts in a paraspeckle is limited by the free energy $F_{\text{shl}}$ of blocks in the shell and the stretching free energy $F_{\text{str}}$ of blocks in the core, analogous to micelles of diblock copolymers. These free energy contributions decreases as the fraction α of A blocks in the shell decreases. Indeed, the fraction α of A blocks in the shell decreases with decreasing the number $N_{\text{A}}$ of units in the A blocks, see Figure 6C. More transcripts can therefore associate with paraspeckles as the number $N_{\text{A}}$ of units in A the blocks decreases. These predictions are consistent with our recent experiments, see Discussion and Yamazaki et al., 2021.

### Paraspeckles are assembled by the association of the middle regions

**FIGURE 7:**
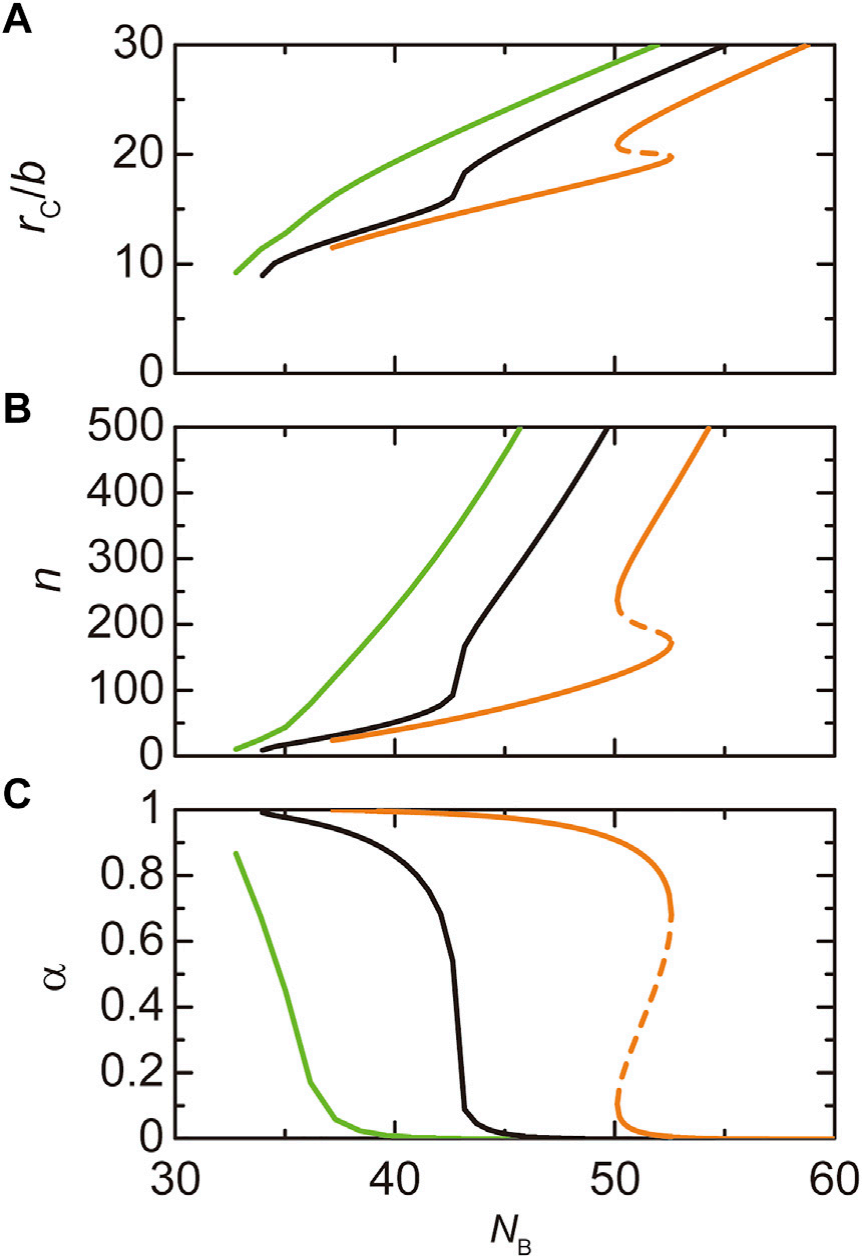
The radius $r_{\text{C}}/b$ **(A)**, the number n of transcripts **(B)**, and the fraction α of the A blocks in the shell **(C)** of a paraspeckle is shown as functions of the number $N_{\text{B}}$ of units in the B blocks for cases in which the number $N_{\text{A}}$ of units in the A blocks is 3.0 (light green), 5.33231 (black), and 8.0 (orange). The (natural) logarithm $\log\left(k_{\text{tx}}/k_{0}\right)$ of the transcription rate is fixed to −12.0. The values of parameters used for the calculations are $N_{\text{C}}$ = 15.0, $\chi_{\text{B}}$ = 0.5, $\chi_{AB}$ = 1.0, $v_{A}/b^{3}=v_{C}/b^{3}$ = 1.0, and $C_{\text{s}}$ = 1.5.

**FIGURE 8:**
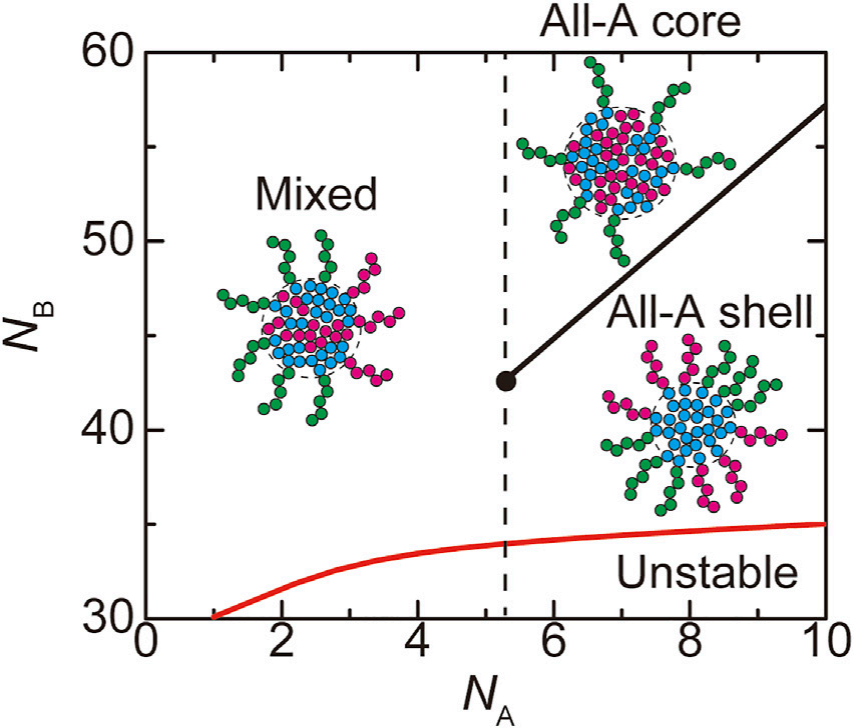
The phase diagram of paraspeckles is shown for the number $N_{\text{B}}$ of units in the B blocks and the number $N_{A}$ of units in the A blocks. The values of parameters used for the calculations are $\log\left(k_{\text{tx}}/k_{0}\right)$ = −12.0, $N_{\text{C}}$ = 15.0, $\chi_{\text{B}}$ = 0.5, $\chi_{\text{AB}}$ = 1.0, $v_{A}/b^{3}=v_{C}/b^{3}$ = 1.0 and $C_{\text{s}}$ = 1.5. The vertical broken line indicates the critical number $N_{\text{Ac}}$ of units in the A blocks.

Paraspeckles are assembled by the attractive interactions between the B blocks of NEAT1_2 RNP complexes. As expected, the radius $r_{\text{C}}$ of paraspeckles decreases as the number $N_{\text{B}}$ of units in the B blocks decreases, see Figure 7A. With a fixed transcription rate, the number n of transcripts in the paraspeckle decreases with decreasing the number $N_{\text{B}}$ of units in the B blocks. It is because decreasing the number $N_{\text{B}}$ of units in the B blocks increases the density of A and C blocks in the shell and thus increases the free energy $F_{\text{shl}}$ of A and C blocks in the shell, see Figure 7B. It also increases the extent of the stretching of B blocks in the core and thus increases the stretching free energy $F_{\text{str}}$ of the B blocks in the core. The fraction α of A blocks in the shell increases as the number $N_{\text{B}}$ of units in B blocks decreases because A blocks in the core suppress the attractive interactions between the B blocks, see Figure 7C. The formation of paraspeckles is suppressed for cases in which the number $N_{\text{B}}$ of units in the B blocks is too small, see Figure 8. These predictions are consistent with our experiments that show that paraspeckles of mutant cells, in which a part of the middle region of NEAT1_2 is deleted, are small and dispersed, compared with the WT, see also the Discussion and Yamazaki et al., 2018.

## Discussion

We have constructed a theory of paraspeckle assembly by taking into account the transcription dynamics of NEAT1_2 in an extension of the theory of ABC triblock copolymer micelles. This model captures two features of paraspeckles: paraspeckles form the characteristic core-shell structure and the assembly of paraspeckles is coupled with the transcription of NEAT1_2. Our theory provides a biophysical insight into the assembly of paraspeckles. Paraspeckles are assembled by multivalent interactions between the middle blocks of the NEAT1_2 RNP complexes (Yamazaki et al., 2018). The repulsive interactions between A and B blocks, quantified by the interaction free energy $F_{\text{AB}}$ , expels A blocks from the core, while the thermal fluctuations, quantified by the mixing free energy $F_{\text{mix}}$ , distribute A blocks randomly between the core and the shell. All the A blocks are localized in the shell for the WT, where the number *N*_A_ of units in A block is large enough, because the interaction free energy $F_{\text{AB}}$ dominates the mixing free energy $F_{\text{mix}}$ . This reflects the connectivity of RBPs *via* the terminal regions of NEAT1_2 and is a well-known feature of polymeric molecules (Doi 1996). This also accounts for the fact that the all-A shell structure of WT paraspeckles is not sensitive to the upregulation of transcription, even for spherical paraspeckles.

**FIGURE 9:**
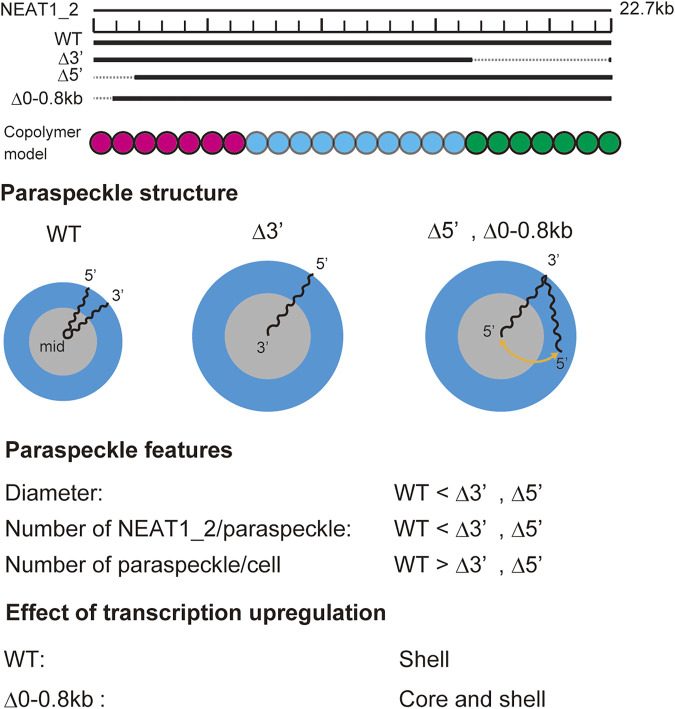
Summary of experimental results on paraspeckles in wild type and deletion mutants (Yamazaki et al., 2021). Copolymer model is schematic and the borders of A, B, and C blocks remain to be experimentally characterized.

Our recent experiments have revealed that (I) the 5′ terminal regions of NEAT1_2 transcripts were distributed randomly between the shell and the core in paraspeckles of Δ5′ deletion mutants, where the 5′ terminal region of NEAT1_2 was deleted by ≈ 2 kb, (II) the 3′ terminal region of most NEAT1_2 transcripts was localized in the core of paraspeckles of Δ3′ deletion mutants, where the 3′ terminal region of NEAT1_2 was deleted by 6 kb, (III) the sizes of paraspeckles in Δ5′ and Δ3′ deletion mutants were larger than the size of wild type paraspeckles, (IV) paraspeckles of deletion mutant, where the middle region of NEAT1_2 was deleted by 8.6 kb, were smaller and more dispersed than the wild type paraspeckles, (V) in deletion mutants, the fraction of the terminal blocks localized in the shell decreased when the transcription of NEAT1_2 was upregulated (Yamazaki et al., 2018, 2021). The core-shell structure of paraspeckles is disorganized in Δ5′- Δ3′ double deletion mutants (Yamazaki et al., 2018, 2021). The assembly of such disordered paraspeckles has been studied theoretically in our recent research (Yamamoto et al., 2020). The summary of these results is shown in Figure 9. The predictions of our theory are consistent with these experimental results. The assembly of paraspeckles can be therefore viewed as the micellization of NEAT1_2 RNP complexes, at least in the first approximation. More quantitative comparison requires the characterization of the interaction parameters between each pair of units by, for example, osmotic pressure measurements (Mangenot et al., 2002) and scattering techniques (Oohashi et al., 2014).

Our theory provides a biophysical insight into our experimental results: In the WT, all the A blocks are localized in the shell because the free energy $F_{\text{AB}}$ due to the repulsive interaction between A and B blocks dominates the mixing free energy $F_{\text{mix}}$ , see the first paragraph of this Discussion. However, the mixing free energy $F_{\text{mix}}$ becomes significant as the number $N_{\text{A}}$ of units in A blocks decreases. Moreover, the excluded volume interactions between A blocks and those between C blocks, quantified by the free energy $F_{\text{shl}}$ , as well as the stretching of B blocks in the core, quantified by the free energy $F_{\text{str}}$ , drive the relocation of A blocks from the shell to the core. This explains our experimental results (I) and (II), see Figure 9. Because these free energy contributions, $F_{\text{shl}}$ and $F_{\text{str}}$ , limit the number of NEAT1_2 transcripts that form paraspeckles, the paraspeckles of deletion mutant can accommodate more NEAT1_2 transcripts. The size of paraspeckles can increase with decreasing the length of the terminal regions of NEAT1_2 because the number of NEAT1_2 transcripts in a paraspeckle increases, see Figure 9. In contrast, the number of NEAT 1_2 transcripts decreases with decreasing the length of the B blocks because the free energy decreases due to the attractive interactions between B blocks by the association of NEAT1_2 RNP complexes becomes less significant than the free energy increase due to the repulsive interactions between A blocks and those between C blocks. The radius of paraspeckles thus decreases as the number $N_{\text{B}}$ of units in B blocks decreases, see Figure 9. The number n . of NEAT1_2 in a paraspeckle increases with the upregulation of the transcription of NEAT1_2. The free energy contributions, $F_{\text{shl}}$ and $F_{\text{str}}$ , increase relative to the free energy with increasing the number of NEAT1_2 in a paraspeckle $F_{\text{AB}}$ . This drives the relocation of A blocks from the shell to the core with the upregulation of NEAT1_2 transcription in the deletion mutant. The all-A shell configuration of a WT paraspeckle does not change with the upregulation of NEAT1_2 transcription, see the first paragraph of this Discussion.

In our approach, we take into account only the essential features to understand the mechanism of the assembly of paraspeckles, instead of doing computer simulations by including all known things. It is indeed the strategy of theoretical physics. Our theory is certainly an important first step, but there are a couple of remaining mysteries. First, we focused on the analysis of spherical paraspeckles, not to hide the essence of the assembly of paraspeckles behind the complexity in treating cylindrical paraspeckles. We thus did not explain the sphere-cylinder morphological transition of paraspeckles. The fact that the fraction of cylindrical paraspeckles increases with the upregulation of NEAT1_2 transcription is indeed consistent with our conclusion that paraspeckles are assembled by micellization. Second, in the WT, 3′ and 5’ terminal regions are not randomly mixed, but are separated in microdomains (West et al., 2016). Our present theory takes into account this feature in the free energy $F_{\text{shl}}$ of the shell by neglecting the interaction between A and C blocks, but did not explain it theoretically. Identifying the RBPs bound to A and C blocks will greatly help to understand the mechanism of the assembly of the microdomains. One possible explanation of the assembly of microdomains is that RBPs bound to A and C are different and the interactions between different blocks are more repulsive than the interactions between the same blocks. However, the microdomains are probably assembled by the microphase separation, judging from the fact that multiple microdomains at the shell of a paraspeckle do not show fusion, and the A-C interaction alone is not enough to explain the assembly of the microdomains. The super-resolution microscope experiments suggest that multiple NEAT1_2 RNPs form a bundle (West et al., 2016). This bundling probably plays an important role in the assembly of the microdomains of A and C blocks. Third, we used the steady state approximation to derive the distribution of the number n of NEAT1_2 transcripts in a paraspeckle. However, the number of NEAT1_2 transcripts that can be incorporated in a paraspeckle should be limited by the number of NEAT1_2 transcripts produced in one transcription burst. The experiments that study the relationship between the transcription dynamics and the number of NEAT1_2 transcripts per paraspeckles would greatly help our understanding of the assembly mechanism of paraspeckles.

## Conclusion

By the combination of theory and experiments, we have shown that paraspeckles are assembled by micellization, not liquid-liquid phase separation. One important feature of micelles is that their size is regulated by the balance between the surface free energy $F_{\text{sur}}$ (that drives the growth of micelles) and the free energy $F_{\text{shl}}$ due to the repulsive interactions between blocks in the shell (that limits the growth of micelles). The free energy $F_{\text{str}}$ due to the entropic elasticity of blocks in the core also limits the growth of micelles. The number of paraspeckles per cell is usually larger than the number of the transcription sites of NEAT1, implying that paraspeckles can diffuse far away from the transcription sites. Paraspeckles may take advantage of this size control mechanism to gain the mobility toward target sites through the meshwork of chromatin in a nucleus.

Our theory and experiments provide insight into the general principle of the assembly of nuclear bodies: The pattern of RBPs binding to arcRNAs is tailored into their RNA sequences and the RNP complexes behave as copolymers that direct the ordering in the internal structures of nuclear bodies. Our theory can be therefore extended to understand the mechanism of the assembly of other nuclear bodies by using the experimentally determined arrangement of RBPs along arcRNAs. The assembly of nuclear bodies is facilitated by the transcription of arcRNAs. The number of arcRNAs per nuclear body is controlled by both the interactions and connectivity of RBPs bound to the arcRNAs and the transcription dynamics. Chemical engineers control the stability and size of liquid condensates by using surfactants. Life takes the same strategy by using RNA.

## Data Availability

The datasets presented in this study can be found in online repositories. The names of the repository/repositories and accession number(s) can be found below: https://doi.org/10.6084/m9.figshare.20209202.

## References

[B1] Alexander S. (1977). Adsorption of chain molecules with a polar head a scaling description. J. Phys. Fr. 28, 983–987. 10.1051/jphys:01977003808098300 10.1051/jphys:01977003808098300 | Google Scholar

[B2] Annianson E. A. G. Wall S. N. (1974). On the kinetics of step-wise micelle association. J. Phys. Chem. 78, 1024–1030. 10.1021/j100603a016 10.1021/j100603a016 | Google Scholar

[B3] Banani S. F. Lee H. O. Hyman A. A. Rosen M. K. (2017). Biomolecular condensates: Organizers of cellular biochemistry. Nat. Rev. Mol. Cell. Biol. 18, 285–298. 10.1038/nrm.2017.7 PubMed Abstract | 10.1038/nrm.2017.7 | Google Scholar 28225081 PMC7434221

[B4] Berry J. Weber S. C. Vaidya N. Haataja M. Brangwynne C. P. (2015). RNA transcription modulates phase transition-driven nuclear body assembly. Proc. Natl. Acad. Sci. U. S. A. 112, E5237–E5245. 10.1073/pnas.1509317112 PubMed Abstract | 10.1073/pnas.1509317112 | Google Scholar 26351690 PMC4586886

[B5] Bonetti A. Agostini F. Suzuki A. M. Hashimoto K. Pascarella G. Gimenez J. (2020). RADICL-seq identifies general and cell type–specific principles of genome-wide RNA-chromatin interactions. Nat. Commun. 11, 1018. 10.1038/s41467-020-14337-6 PubMed Abstract | 10.1038/s41467-020-14337-6 | Google Scholar 32094342 PMC7039879

[B6] Borisov O. V. Halperin A. (1995). Micelles of polysoaps. Langmuir 11, 2911–2919. 10.1021/la00008a012 10.1021/la00008a012 | Google Scholar

[B7] Cai Z. Cao C. Ji L. Ye R. Wang D. Xia C. (2020). RIC-seq for global *in situ* profiling of RNA-RNA spatial interactions. Nature 582, 432–437. 10.1038/s41586-020-2249-1 PubMed Abstract | 10.1038/s41586-020-2249-1 | Google Scholar 32499643

[B8] Chen L. L. Carmichael G. G. (2009). Altered nuclear retention of mRNAs containing inverted repeats in human embryonic stem cells: Functional role of a nuclear noncoding RNA. Mol. Cell. 35, 467–478. 10.1016/j.molcel.2009.06.027 PubMed Abstract | 10.1016/j.molcel.2009.06.027 | Google Scholar 19716791 PMC2749223

[B9] Chujo T. Hirose T. (2017). Nuclear bodies built on architectural long noncoding RNAs: Unifying principles of their construction and function. Mol. Cells 40, 889–896. 10.14348/molcells.2017.0263 PubMed Abstract | 10.14348/molcells.2017.0263 | Google Scholar 29276943 PMC5750707

[B10] Chujo T. Yamazaki T. Hirose T. (2016). Architectural RNAs (arcRNAs): A class of long noncoding RNAs that function as the scaffold of nuclear bodies. Biochim. Biophys. Acta 1859, 139–146. 10.1016/j.bbagrm.2015.05.007 PubMed Abstract | 10.1016/j.bbagrm.2015.05.007 | Google Scholar 26021608

[B11] Clemson C. M. Hutchinson J. N. Sara S. A. Ensminger A. W. Fox A. H. Chess A. (2009). An architectural role for a nuclear noncoding RNA: *NEAT1* RNA is essential for the structure of paraspeckles. Mol. Cell. 33, 717–726. 10.1016/j.molcel.2009.01.026 PubMed Abstract | 10.1016/j.molcel.2009.01.026 | Google Scholar 19217333 PMC2696186

[B12] Daoud M. Cotton J. P. (1982). Star shaped polymers: A model for the conformation and its concentration dependence. J. Phys. Fr. 43, 531–538. 10.1051/jphys:01982004303053100 10.1051/jphys:01982004303053100 | Google Scholar

[B13] de Gennes P. G. (1980). Conformations of polymers attached to an interface. Macromolecules 13, 1069–1075. 10.1021/ma60077a009 10.1021/ma60077a009 | Google Scholar

[B14] Doi M. (1996). Introduction to polymer physics. New York, USA: Oxford Univ. Press. Google Scholar

[B15] Fang J. Ma Q. Chu C. Huang B. Li L. Cai P. (2019). PIRCh-seq: Functional classification of non-coding RNAs associated with distinct histone modifications. Genome Biol. 20, 292. 10.1186/s13059-019-1880-3 PubMed Abstract | 10.1186/s13059-019-1880-3 | Google Scholar 31862000 PMC6924075

[B16] Hadgiivanova R. Diamant H. Andelman D. (2011). Kinetics of surfactant micellization: A free energy approach. J. Phys. Chem. B 115, 7268–7280. 10.1021/jp1073335 PubMed Abstract | 10.1021/jp1073335 | Google Scholar 21158411

[B17] Halperin A. Alexander S. (1989). Polymeric micelles: Their relaxation kinetics. Macromolecules 22, 2403–2412. 10.1021/ma00195a069 10.1021/ma00195a069 | Google Scholar

[B18] Hirose T. Virnicchi G. Tanigawa A. Naganuma T. Li R. Kimura H. (2014). NEAT1 long noncoding RNA regulates transcription via protein sequestration within subnuclear bodies. Mol. Biol. Cell. 25, 169–183. 10.1091/mbc.E13-09-0558 PubMed Abstract | 10.1091/mbc.E13-09-0558 | Google Scholar 24173718 PMC3873887

[B19] Hu S. B. Xiang J. F. Li X. Xu Y. Xue W. Huang M. (2015). Protein arginine methyltransferase CARM1 attenuates the paraspeckle-mediated nuclear retention of mRNAs containing IRAlus. Genes Dev. 29, 630–645. 10.1101/gad.257048.114 PubMed Abstract | 10.1101/gad.257048.114 | Google Scholar 25792598 PMC4378195

[B20] Imamura K. Imamachi N. Akizuki G. Kumakura M. Kawaguchi A. Nagata K. (2014). Long noncoding RNA NEAT1-dependent SFPQ relocation from promoter region to paraspeckle mediates IL8 expression upon immune stimuli. Mol. Cell. 53, 393–406. 10.1016/j.molcel.2014.01.009 PubMed Abstract | 10.1016/j.molcel.2014.01.009 | Google Scholar 24507715

[B21] Li X. Zhou B. Chen L. Gou L. T. Li H. Fu X. D. (2017). GRID-seq reveals the global RNA-chromatin interactome. Nat. Biotechnol. 35, 940–950. 10.1038/nbt.3968 PubMed Abstract | 10.1038/nbt.3968 | Google Scholar 28922346 PMC5953555

[B22] Mai Y. Eisenberg A. (2012). Self-assembly of block copolymers. Chem. Soc. Rev. 41, 5969–5985. 10.1039/c2cs35115c PubMed Abstract | 10.1039/c2cs35115c | Google Scholar 22776960

[B23] Mangenot S. Raspaud E. Tribet C. Belloni L. Livolant F. (2002). Interactions between isolated nucleosome core particles: A tail-bridging effect? Eur. Phys. J. E 7, 221–231. 10.1140/epje/i200101151 10.1140/epje/i200101151 | Google Scholar

[B24] Mao Y. S. Sunwoo H. Zhang B. Spector D. L. (2011). Direct visualization of the co-transcriptional assembly of a nuclear body by noncoding RNAs. Nat. Cell. Biol. 13, 95–101. 10.1038/ncb2140 PubMed Abstract | 10.1038/ncb2140 | Google Scholar 21170033 PMC3007124

[B25] Monzen M. Kawakatsu T. Doi M. (2000). Micelle formation in triblock copolymer solutions. Comput. Theor. Polym. Sci. 10, 275–280. 10.1016/s1089-3156(99)00052-5 10.1016/s1089-3156(99)00052-5 | Google Scholar

[B26] Moughton A. Hillmyer M. A. Lodge T. P. (2012). Multicompartment block polymer micelles. Macromolecules 45, 2–19. 10.1021/ma201865s 10.1021/ma201865s | Google Scholar

[B27] Mysona J. A. McCormick A. V. Morse D. C. (2019). Mechanism of micelle birth and death. Phys. Rev. Lett. 123, 038003. 10.1103/PhysRevLett.123.038003 PubMed Abstract | 10.1103/PhysRevLett.123.038003 | Google Scholar 31386437

[B28] Nakagawa S. Yamazaki T. Hirose T. (2018). Molecular dissection of nuclear paraspeckles: Towards understanding the emerging world of the RNP milieu. Open Biol. 8, 180150. 10.1098/rsob.180150 PubMed Abstract | 10.1098/rsob.180150 | Google Scholar 30355755 PMC6223218

[B29] Netz R. R. Schick M. (1998). Polymer brushes: From self-consistent field theory to classical theory. Macromolecules 31, 5105–5122. 10.1021/ma9717505 PubMed Abstract | 10.1021/ma9717505 | Google Scholar 9680451

[B30] Oohashi T. Inoue K. Nakamura Y. (2014). Second and third virial coefficients of low-molecular-weight polyisoprene in 1, 4-dioxane. Polym. J. 46, 699–703. 10.1038/pj.2014.43 10.1038/pj.2014.43 | Google Scholar

[B31] Palikyaras S. Papantonis A. (2019). Modes of phase separation affecting chromatin regulation. Open Biol. 9, 190167. 10.1098/rsob.190167 PubMed Abstract | 10.1098/rsob.190167 | Google Scholar 31615334 PMC6833219

[B32] Safran S. A. (2003). Statistical thermodynamics of surfaces, interfaces, and membranes. USA: Westview Press, CO. Google Scholar

[B33] Sasaki Y. T. F. Ideue T. Sano M. Mituyama T. Hirose T. (2009). MENepsilon/beta noncoding RNAs are essential for structural integrity of nuclear paraspeckles. Proc. Natl. Acad. Sci. U. S. A. 106, 2525–2530. 10.1073/pnas.0807899106 PubMed Abstract | 10.1073/pnas.0807899106 | Google Scholar 19188602 PMC2650297

[B34] Semenov A. N. (1985). Contribution to the theory of microphase layering in block-copolymer melts. Sov. Phys. JETP 61, 733–742. http://www.jetp.ras.ru/cgi-bin/e/index/e/61/4/p733?a=list. Google Scholar

[B35] Semenov A. N. Nyrkova I. A. Khokhlov A. R. (1995). Polymers with strongly interacting Groups: Theory for nonspherical multiplets. Macromolecules 28, 7491–7500. 10.1021/ma00126a029 10.1021/ma00126a029 | Google Scholar

[B36] Shin Y. Brangwynne C. P. (2017). Liquid phase condensation in cell physiology and disease. Science 357, eaaf4382. 10.1126/science.aaf4382 PubMed Abstract | 10.1126/science.aaf4382 | Google Scholar 28935776

[B37] Souquere S. Beauclair G. Harper F. Fox A. Pierron G. (2010). Highly ordered spatial organization of the structural long noncoding Neat1 RNAs within paraspeckle nuclear bodies. Mol. Biol. Cell. 21, 4020–4027. 10.1091/mbc.E10-08-0690 PubMed Abstract | 10.1091/mbc.E10-08-0690 | Google Scholar 20881053 PMC2982136

[B38] Sridhar B. Rivas-Astroza M. Nguyen T. C. Chen W. Yan Z. Cao X. (2017). Systematic mapping of RNA-chromatin interactions *in vivo* . Curr. Biol. 20, 602–609. 10.1016/j.cub.2017.01.011 10.1016/j.cub.2017.01.011 | Google Scholar PMC5319903 28132817

[B39] Sunwoo H. Dinger M. E. Wilusz J. E. Amaral P. P. Mattick J. S. Spector D. L. (2008). MEN epsilon/beta nuclear-retained non-coding RNAs are up-regulated upon muscle differentiation and are essential components of paraspeckles. Genome Res. 19, 347–359. 10.1101/gr.087775.108 PubMed Abstract | 10.1101/gr.087775.108 | Google Scholar 19106332 PMC2661813

[B40] Wang Y. Hu S. B. Wang M. R. Yao R. W. Wu D. Yang L. (2018). Genome-wide screening of NEAT1 regulators reveals cross-regulation between paraspeckles and mitochondria. Nat. Cell. Biol. 20, 1145–1158. 10.1038/s41556-018-0204-2 PubMed Abstract | 10.1038/s41556-018-0204-2 | Google Scholar 30250064

[B41] West J. A. Davis C. P. Sunwoo H. Simon M. D. Sadreyev R. Wang P. I. (2014). The long noncoding RNAs NEAT1 and MALAT1 bind active chromatin sites. Mol. Cell. 55, 791–802. 10.1016/j.molcel.2014.07.012 PubMed Abstract | 10.1016/j.molcel.2014.07.012 | Google Scholar 25155612 PMC4428586

[B42] West J. A. Mito M. Kurosaka S. Takumi T. Tanegashima C. Chujo T. (2016). Structural, super-resolution microscopy analysis of paraspeckle nuclear body organization. J. Cell. Biol. 214, 817–830. 10.1083/jcb.201601071 PubMed Abstract | 10.1083/jcb.201601071 | Google Scholar 27646274 PMC5037409

[B43] Yamamoto T. Yamazaki T. Hirose T. (2020). Phase separation driven by production of architectural RNA transcripts. Soft Matter 16, 4692–4698. 10.1039/c9sm02458a PubMed Abstract | 10.1039/c9sm02458a | Google Scholar 32396591

[B44] Yamazaki T. Nakagawa S. Hirose T. (2020). Architectural RNAs for membraneless nuclear body formation. Cold Spring Harb. Symp. Quant. Biol. 84, 227–237. 10.1101/sqb.2019.84.039404 PubMed Abstract | 10.1101/sqb.2019.84.039404 | Google Scholar 32019862

[B45] Yamazaki T. Souquere S. Chujo T. Kobelke S. Chong Y. S. Fox A. H. (2018). Functional domains of NEAT1 architectural lncRNA induce paraspeckle assembly through phase separation. Mol. Cell. 70, 1038–1053. 10.1016/j.molcel.2018.05.019 PubMed Abstract | 10.1016/j.molcel.2018.05.019 | Google Scholar 29932899

[B46] Yamazaki T. Yamamoto T. Yoshino H. Souquere S. Nakagawa S. Pierron G. (2021). Paraspeckles are constructed as block copolymer micelles. EMBO J. 40, e107270. 10.15252/embj.2020107270 PubMed Abstract | 10.15252/embj.2020107270 | Google Scholar 33885174 PMC8204865

[B47] Yap K. Chung T. H. Makeyev E. V. (2022). Hybridization-proximity labeling reveals spatially ordered interactions of nuclear RNA compartments. Mol. Cell. 82, 463–478.e11. 10.1016/j.molcel.2021.10.009 PubMed Abstract | 10.1016/j.molcel.2021.10.009 | Google Scholar 34741808 PMC8791277

[B48] Zhulina E. B. Adam M. LaRue I. Sheiko S. S. Rubinstein M. (2005). Diblock copolymer micelles in a dilute solution. Macromolecules 38, 5330–5351. 10.1021/ma048102n 10.1021/ma048102n | Google Scholar

[B49] Zhulina E. B. Birshtein T. M. Borisov O. V. (2006). Curved polymer and polyelectrolyte brushes beyond the Daoud-Cotton model. Eur. Phys. J. E Soft Matter 20, 243–256. 10.1140/epje/i2006-10013-5 PubMed Abstract | 10.1140/epje/i2006-10013-5 | Google Scholar 16862399

