# Supplementary material for: Triblock copolymer micelle model of spherical paraspeckles
Source: Front Mol Biosci. 2022 Aug 22;9:925058. doi: 10.3389/fmolb.2022.925058 (PMC9441768; doi:10.3389/fmolb.2022.925058)
Supplement: Supplementary file 1 [file DataSheet1.PDF]

frontiers

Supplementary Material

S1 FREE ENERGY DUE TO CONFORMATIONAL ENTROPY OF B BLOCKS

The free energy due to the conformational entropy of blocks in the core was derived by Semenov in the limit of strong segregation (Semenov (1985)). This theory treats the core as a spherical melt brush. We here use the same approach to analyze the free energy due to the conformational entropy of the B blocks in the core of a paraspeckle. In a paraspeckle, the B blocks of copolymers that have their A blocks in the shell form loops, whereas the B blocks of copolymers that have their A blocks in the core are straight in the core. The free energy due to the conformational entropy has the form

$$\begin{aligned}\frac{F_{\mathrm{c}}}{k_{\mathrm{B}}T} &= \frac{3}{2b^2}\int_0^{r_{\mathrm{c}}} dr_0 \int_0^{r_0} dr\, E_{\mathrm{s}}(r,r_0)g_{\mathrm{s}}(r_0) \\ &\quad + \frac{3}{b^2}\int_0^{r_{\mathrm{c}}} dr_0 \int_0^{r_0} dr E_{\mathrm{l}}(r,r_0)g_{\mathrm{l}}(r_0), \end{aligned} \tag{S1}$$

where $E_{\mathrm{s}}(r,r_0) = |dr_{\mathrm{s}}(n,r_0)/dn|$ and $E_{\mathrm{l}}(r,r_0) = |dr_{\mathrm{l}}(n,r_0)/dn|$ are the local stretching of the straight and loop blocks. r is the distance from the interface between the core and the shell. $g_{\mathrm{s}}(r_0)$ is the distribution functions of the free end of the straight blocks and $g_{\mathrm{l}}(r_0)$ is the distribution function of the middle unit of the loop blocks. In eq. (S1), we assume that the most probable path of a loop is symmetric with respect to the unit in the middle of the B block.

Eq. (S1) should be minimized with respect to $E_{\mathrm{s}}(r,r_0)$ and $E_{\mathrm{l}}(r,r_0)$ with the constraints due to the fact that all blocks have the same the number of units

$$\int_0^{r_0} \frac{dr}{E_{\mathrm{s}}(r,r_0)} = N_{\mathrm{A}} + N_{\mathrm{B}} \tag{S2}$$

$$2\int_0^{r_0} \frac{dr}{E_{\mathrm{l}}(r,r_0)} = N_{\mathrm{B}} \tag{S3}$$

and the incompressibility condition

$$\int_r^{r_{\mathrm{c}}} dr_0 \frac{g_{\mathrm{s}}(r_0)}{E_{\mathrm{s}}(r,r_0)} + 2\int_r^{r_{\mathrm{c}}} dr_0 \frac{g_{\mathrm{l}}(r_0)}{E_{\mathrm{l}}(r,r_0)} = \frac{S_d(r_{\mathrm{c}}-r)}{b^d}. \tag{S4}$$

The function $S_d(r)$ depends on the dimensionality d ($d = 3$ for spherical micelles, $d = 2$ for cylindrical micelles, and $d = 1$ for lamellar) and has the form

$$S_1(r) = 2 \tag{S5}$$

$$S_2(r) = 2\pi r \tag{S6}$$

$$S_3(r) = 4\pi r^2. \tag{S7}$$

We take into account these constraints by using the Lagrange multipliers

$$\begin{aligned}\frac{F_{\rm c}}{k_{\rm B}T} &= \frac{3}{2b^2}\int_0^{r_{\rm c}} dr_0 \int_0^{r_0} dr\, E_{\rm s}(r,r_0)g_{\rm s}(r_0)\\ &+\frac{3}{b^2}\int_0^{r_{\rm c}} dr_0 \int_0^{r_0} dr E_{\rm l}(r,r_0)g_{\rm l}(r_0)\\ &+\int_0^{r_{\rm c}} dr_0\mu_{\rm s}(r_0)\int_0^{r_0} dr\frac{1}{E_{\rm s}(r,r_0)}\\ &+2\int_0^{r_{\rm c}} dr_0\mu_{\rm l}(r_0)\int_0^{r_0} dr\frac{1}{E_{\rm l}(r,r_0)}\\ &+\int_0^{r_{\rm c}} dr\Pi_0(r)\int_r^{r_{\rm c}} dr_0\left[\frac{g_{\rm s}(r_0)}{E_{\rm s}(r,r_0)}+\frac{2g_{\rm l}(r_0)}{E_{\rm l}(r,r_0)}\right]. \end{aligned} \tag{S8}$$

Applying the variational principle to eq. (S8) with respect to $E_{\rm s}(r,r_0)$ and $E_{\rm l}(r,r_0)$ leads to the form

$$E_{\rm s}^2(r,r_0) = \frac{2b^2}{3}\left(\Pi_0(r)+\frac{\mu_{\rm s}(r_0)}{g_{\rm s}(r_0)}\right) \tag{S9}$$

$$E_{\rm l}^2(r,r_0) = \frac{2b^2}{3}\left(\Pi_0(r)+\frac{\mu_{\rm l}(r_0)}{g_{\rm l}(r_0)}\right). \tag{S10}$$

The free ends of the A blocks of unlooped chains are not stretched, $E_{\rm s}(r_0,r_0)=0$. The turning units of B blocks of loops are also not stretched, $E_{\rm l}(r_0,r_0)=0$. These boundary conditions lead to the form

$$E_{\rm s}(r,r_0) = (\varphi_{\rm s}(r_0)-\varphi_{\rm s}(r))^{1/2} \tag{S11}$$

$$E_{\rm l}(r,r_0) = (\varphi_{\rm l}(r_0)-\varphi_{\rm l}(r))^{1/2}. \tag{S12}$$

Substituting eqs. (S11) and (S12) into eqs. (S2) and (S3) leads to the forms

$$\int_0^{r_0} dr\frac{1}{(\varphi_{\rm s}(r_0)-\varphi_{\rm s}(r))^{1/2}} = N_{\rm A}+N_{\rm B} \tag{S13}$$

$$2\int_0^{r_0} dr\frac{1}{(\varphi_{\rm l}(r_0)-\varphi_{\rm l}(r))^{1/2}} = N_{\rm B}. \tag{S14}$$

The solutions of eqs. (S13) and (S14) have the forms

$$E_{\rm s}(r,r_0) = \frac{\pi}{2(N_{\rm A}+N_{\rm B})}(r_0^2-r^2)^{1/2} \tag{S15}$$

$$E_{\rm l}(r,r_0) = \frac{\pi}{N_{\rm B}}(r_0^2-r^2)^{1/2}, \tag{S16}$$

where it is useful to refer to Netz and Schick (1998) for the derivation. Substituting eqs. (S15) and (S16) into eq. (S4) leads to the form

$$\frac{2}{\pi}\int_r^{r_{\rm c}} dr_0\frac{(N_{\rm A}+N_{\rm B})g_{\rm s}(r_0)+N_{\rm B}g_{\rm l}(r_0)}{\sqrt{r_0^2-r^2}} = \frac{S_d(r_{\rm c}-r)}{b^d}. \tag{S17}$$

For the case of a spherical paraspeckle ($d = 3$), the solution of eq. (S17) has the form

$$(N_{\mathrm{A}} + N_{\mathrm{B}})g_{\mathrm{s}}(r_0) + N_{\mathrm{B}}g_{\mathrm{l}}(r_0) = N_{\mathrm{B}}g(r_0), \tag{S18}$$

where $g(r_0)$ has the form

$$g(r_0) = \frac{8\pi r_{\mathrm{c}} r_0}{N_{\mathrm{B}} b^3}\left(\tanh^{-1}\sqrt{1 - \frac{r_0^2}{r_{\mathrm{c}}^2}} - \sqrt{1 - \frac{r_0^2}{r_{\mathrm{c}}^2}}\right). \tag{S19}$$

It is useful to refer to Semenov (1985) for the derivation of eq. (S19). The fraction of B blocks that form loops is α,

$$\int_0^{r_0} g_{\mathrm{l}}(r_0) = \alpha. \tag{S20}$$

A simple summation scheme

$$g_{\mathrm{s}}(r_0) = (1 - \alpha)\frac{N_{\mathrm{B}}}{N_{\mathrm{A}} + N_{\mathrm{B}}}g(r_0) \tag{S21}$$

$$g_{\mathrm{l}}(r_0) = \alpha g(r_0) \tag{S22}$$

satisfies both eqs. (S18) and (S20). Substituting eqs. (S21) and (S22) into eq. (S1) leads to the free energy due to the conformational entropy of the B blocks,

$$\frac{F_{\mathrm{c}}}{k_{\mathrm{B}}T} = \frac{3}{2}\lambda_{\mathrm{s}}\frac{r_{\mathrm{c}}^5}{N_{\mathrm{B}}^2 b^2}4\alpha + \frac{3}{2}\lambda_{\mathrm{s}}\frac{r_{\mathrm{c}}^5}{(N_{\mathrm{A}} + N_{\mathrm{B}})^2 b^2}(1 - \alpha), \tag{S23}$$

where λ_{s} $(= \pi^3/30)$ is a constant. The factor 4 in the first term of eq. (S23) reflects the fact that a loop of a B block is viewed as two chains, each composed of $N_{\mathrm{B}}/2$ units.

One criticism of this derivation may be that we used different unknown functions, $\varphi_{\mathrm{s}}(r)$ and $\varphi_{\mathrm{l}}(r)$, for the straight chains and loops in eqs. (S11) and (S12), although both of them are $2b^2\Pi_0(r)/3 + \mathrm{constant}$. Using different functions, $\varphi_{\mathrm{s}}(r)$ and $\varphi_{\mathrm{l}}(r)$, implies that different mean field pressure is applied to the units of straight chains and loops. If we use the same function for the straight chains and loops, it is not possible to satisfy eqs. (S2) and (S3) whatever the choice of $g_{\mathrm{s}}(r_0)$ and $g_{\mathrm{l}}(r_0)$. The solutions, eqs. (S21) and (S22), ensure the constraints, eqs. (S2) - (S4), and thus are at least consistent. Another approach is to divide the core into two layers: one layer at the surface is composed of loops and unlooped chains and the other layer at the center is composed only of unlooped chains. However, we use eq. (S23) for the free energy due to the conformational entropy of B blocks for simplicity.

S2 FREE ENERGY OF SHELL

S2.1 Good solvent

The free energy of the shell has the form

$$F_{\mathrm{sh}} = F_{\mathrm{A}} + F_{\mathrm{C}}, \tag{S24}$$

where $F_{\rm A}$ is the free energy of the A blocks and $F_{\rm C}$ is the free energy of the C blocks. The A and C blocks are not mixed in the shell, but exist in different domains. We therefore neglect the interactions between A and C blocks; the interactions between A and C blocks only occur at the interface between domains.

We here assume that the nucleoplasm is a good solvent for both A and C blocks. In the semi-dilute regime, the scaling theory predicts that the free energy contributions of the A and C blocks are the thermal energy $k_{\rm B}T$ per blob

$$\frac{F_{\rm A}}{k_{\rm B}T} = C_{\rm s}\int_{r_{\rm c}}^{r_{\rm A}} \frac{4\pi r^2 f_{\rm A} dr}{R_{\rm A}^3(r)} \tag{S25}$$

$$\frac{F_{\rm C}}{k_{\rm B}T} = C_{\rm s}\int_{r_{\rm c}}^{r_{\rm C}} \frac{4\pi r^2 (1-f_{\rm A}) dr}{R_{\rm C}^3(r)}, \tag{S26}$$

where $C_{\rm s}$ is a constant of order unity and $C_{\rm s} \simeq 1.38$ was derived by using the experiments on polystylene-polyisoprene copolymer micelles (Zhulina et al. (2005)). $r_{\rm c}$ is the radius of the core. $r_{\rm A}$ is the distance between the center of the core and the top of the A blocks and $r_{\rm C}$ is the distance between the center of the core and the top of the C blocks. $f_{\rm A}$ is the fraction of the surface of the core that is occupied by A blocks. $R_{\rm A}(r)$ and $R_{\rm C}(r)$ are the sizes of the blobs of A and C blocks, respectively. These blob sizes are functions of the distance r from the center of the core.

The sizes, $R_{\rm A}$ and $R_{\rm C}$, of the blobs of A and C blocks have the forms

$$R_{\rm A}(r) = b\left(\frac{v_{\rm A}}{b^3}\right)^{1/5} g_{\rm A}^{3/5}(r) \tag{S27}$$

$$R_{\rm C}(r) = b\left(\frac{v_{\rm C}}{b^3}\right)^{1/5} g_{\rm C}^{3/5}(r)., \tag{S28}$$

where $g_{\rm A}(r)$ is the number of units per A blob and $g_{\rm C}(r)$ is the number of units per C blob. The Daoud-Cotton scaling theory predicts that the sizes, $R_{\rm A}(r)$ and $R_{\rm C}(r)$, of blobs are determined by the average area occupied by the A and C blocks (Daoud and Cotton (1982))

$$4\pi r^2 f_{\rm A} = R_{\rm A}^2(r)\alpha n \tag{S29}$$

$$4\pi r^2(1-f_{\rm A}) = R_{\rm C}^2(r) n. \tag{S30}$$

Eqs. (S27) - (S30) leads to the sizes, $R_{\rm A}$ and $R_{\rm C}$, and the number of units, $g_{\rm A}(r)$ and $g_{\rm C}(r)$, per blob, once the fraction $f_{\rm A}$ is given.

The osmotic pressure of A blocks is $k_{\rm B}T/R_{\rm A}^3(r)$, whereas the osmotic pressure of C blocks is $k_{\rm B}T/R_{\rm C}^3(r)$. The domains of A blocks coexist with the domains of C blocks when the osmotic pressures in these domains are balanced

$$\frac{k_{\rm B}T}{R_{\rm A}^3(r)} = \frac{k_{\rm B}T}{R_{\rm C}^3(r)}, \tag{S31}$$

The fraction $f_{\rm A}$ thus has the form

$$f_{\rm A} = \frac{\alpha}{1+\alpha}. \tag{S32}$$

The distances, $r_{\rm A}$ and $r_{\rm C}$, are derived by the conservation of the number of units

$$\int_{r_{\rm c}}^{r_{\rm A}} \frac{4\pi r^2 f_{\rm A} dr}{R_{\rm A}^3(r)} g_{\rm A}(r) = N_{\rm A}\alpha n \tag{S33}$$

$$\int_{r_{\rm c}}^{r_{\rm C}} \frac{4\pi r^2 (1-f_{\rm A}) dr}{R_{\rm C}^3(r)} g_{\rm C}(r) = N_{\rm C} n. \tag{S34}$$

Eqs. (S33) and (S34) lead to the form

$$r_{\rm A} = r_{\rm c}\left(1+\frac{5}{3}\frac{h_{\rm A}}{r_{\rm c}}\right)^{3/5} \tag{S35}$$

$$r_{\rm C} = r_{\rm c}\left(1+\frac{5}{3}\frac{h_{\rm C}}{r_{\rm c}}\right)^{3/5}, \tag{S36}$$

where $h_{\rm A}$ and $h_{\rm C}$ have the form

$$h_{\rm A} = N_{\rm A} b \left(\frac{n\alpha}{4\pi f_{\rm A} r_{\rm c}^2}\frac{v_{\rm A}}{b}\right)^{1/3} \tag{S37}$$

$$h_{\rm C} = N_{\rm C} b \left(\frac{n}{4\pi(1-f_{\rm A}) r_{\rm c}^2}\frac{v_{\rm C}}{b}\right)^{1/3}. \tag{S38}$$

Eqs. (S37) and (S38) correspond to the heights of planer brushes (note that $\sigma_{\rm A} = n\alpha/(4\pi f_{\rm A} r_{\rm c}^2)$ and $\sigma_{\rm C} = n/(4\pi(1-f_{\rm A})r_{\rm c}^2)$ are the grafting densities of the A and C blocks, respectively). $h_{\rm A}$ and $h_{\rm C}$ are the heights of A and C domains in the limit of $r_{\rm c} \to \infty$, see eqs. (S35) and (S36).

By using eqs. (S27) - (S38), eqs. (S25) and (S26) are rewritten in the forms

$$\frac{F_{\rm A}}{k_{\rm B}T} = \frac{3}{5}\frac{(n\alpha)^{3/2}}{(4\pi f_{\rm A})^{1/2}} C_{\rm s} \log\left(1+\frac{5}{3}\frac{h_{\rm A}}{r_{\rm c}}\right) \tag{S39}$$

$$\frac{F_{\rm C}}{k_{\rm B}T} = \frac{3}{5}\frac{n^{3/2}}{(4\pi(1-f_{\rm A}))^{1/2}} C_{\rm s} \log\left(1+\frac{5}{3}\frac{h_{\rm C}}{r_{\rm c}}\right). \tag{S40}$$

S2.2 Marginal solvent

For cases in which the excluded volumes, $v_{\rm A}$ and $v_{\rm C}$, are small (marginal solvent), the free energy is derived by the mean field theory. With the mean field theory, the free energy of the A blocks has the form

$$\frac{F_{\rm A}}{k_{\rm B}T} = \int_{r_{\rm c}}^{r_{\rm A}} \frac{4\pi r^2 f_{\rm A} dr}{R_{\rm A}^3}\left[\frac{3}{2}\frac{R_{\rm A}^2(r)}{b^2 g_{\rm A}(r)} + v_{\rm A}\frac{g_{\rm A}^2(r)}{R_{\rm A}^3(r)}\right], \tag{S41}$$

$$\frac{F_{\rm C}}{k_{\rm B}T} = \int_{r_{\rm c}}^{r_{\rm C}} \frac{4\pi r^2 (1-f_{\rm A}) dr}{R_{\rm C}^3}\left[\frac{3}{2}\frac{R_{\rm C}^2(r)}{b^2 g_{\rm C}(r)} + v_{\rm C}\frac{g_{\rm C}^2(r)}{R_{\rm C}^3(r)}\right]. \tag{S42}$$

$R_{\rm A}(r)$ and $R_{\rm C}(r)$ are the sizes of blobs of A and C blocks at a distance r from the center of the spherical paraspeckle, see eqs. (S29) and (S30). $g_{\rm A}(r)$ and $g_{\rm C}(r)$ are the number of units in each blob of A and C blocks, see eqs. (S27) and (S28). $r_{\rm c}$ is the radius of the core. $r_{\rm A}$ is the distance between the top of the A

blocks and the center of the core, see eq. (S35). $r_{\rm C}$ is the distance between the top of the C blocks and the center of the core, see eq. (S36). $f_{\rm A}$ is the fraction of the interfacial area occupied by the A blocks, where its form is different from the good solvent regime. $v_{\rm A}$ and $v_{\rm C}$ is the excluded volumes of A and C units. $4\pi r^2 f_{\rm A}(r) dr/R_{\rm A}^3$ and $4\pi r^2(1-f_{\rm A}(r))dr/R_{\rm C}^3$ is the number of blobs of A and C blocks in the spherical shell of thickness dr at a distance r from the center of the core. The terms inside the square bracket is the free energy of each blob and has the form of the free energy of the Flory theory of swollen chains.

The fraction $f_{\rm A}$ of the interfacial area occupied by the A blocks is derived by the balance of the osmotic pressure between the A blocks and the C blocks in the shell,

$$v_{\rm A}\frac{g_{\rm A}^2(r)}{R_{\rm A}^6(r)} = v_{\rm C}\frac{g_{\rm C}^2(r)}{R_{\rm C}^6(r)}. \tag{S43}$$

Eqs. (S27) - (S43) leads to the form

$$f_{\rm A} = \frac{\alpha v_{\rm A}^{1/4}}{\alpha v_{\rm A}^{1/4} + v_{\rm C}^{1/4}} \tag{S44}$$

and is not a function of the distance r.

By using eqs. (S27) - (S38), eqs. (S41) and (S42) are rewritten in the form

$$\frac{F_{\rm A}}{k_{\rm B}T} = \frac{15}{2}\alpha n \frac{h_{\rm A}}{bN_{\rm A}}\frac{r_{\rm c}}{b}\left[\left(1+\frac{5}{3}\frac{h_{\rm A}}{r_{\rm c}}\right)^{1/5} - 1\right] \tag{S45}$$

$$\frac{F_{\rm C}}{k_{\rm B}T} = \frac{15}{2} n \frac{h_{\rm C}}{bN_{\rm C}}\frac{r_{\rm c}}{b}\left[\left(1+\frac{5}{3}\frac{h_{\rm C}}{r_{\rm c}}\right)^{1/5} - 1\right]. \tag{S46}$$

In the limit of large $r_{\rm c}$, eqs. (S45) and (S46) have approximate forms

$$\frac{F_{\rm A}}{k_{\rm B}T} = \frac{5}{2}\alpha n \frac{h_{\rm A}^2}{b^2 N_{\rm A}} \tag{S47}$$

$$\frac{F_{\rm C}}{k_{\rm B}T} = \frac{5}{2} n \frac{h_{\rm C}^2}{b^2 N_{\rm C}}, \tag{S48}$$

which are the forms of the free energy of planar brushes.

S3 SUPPLEMENTARY DISCUSSION

Both the free energy due to the excluded volume interactions between A units in the shell and the stretching free energy of blocks in the core decreases the fraction α of A blocks in the shell and limit the number n of transcripts in a paraspeckle. The free energy of A blocks in the shell scales as $N_{\rm A} N_{\rm B}^{-5/9} n^{23/18}$ and the stretching free energy of blocks in the core scales as $N_{\rm B}^{-1/3} n^{5/3}$ for cases in which the number n of transcripts in the paraspeckle is large and $N_{\rm B} > N_{\rm A}$ (see the first term of eq. (2) and eq. (6) in MATERIALS AND METHODS in the main article). The free energy of A blocks in the shell dominates for cases in which the number n of transcripts in the paraspeckle is small, $n < N_{\rm A}^{18/7} N_{\rm B}^{-4/7}$,

whereas the stretching free energy of blocks in the core dominates for cases in which the number n of transcripts in the paraspeckle is large, $n > N_{\mathrm{A}}^{18/7} N_{\mathrm{B}}^{-4/7}$.

REFERENCES

Daoud, M. and Cotton, J. (1982). Star shaped polymers: a model for the conformation and its concentration dependence. *J. Phys. France* 43, 531–538

Netz, R. and Schick, M. (1998). Polymer brushes: From self-consistent field theory to classical theory. *Macromolecules* 31, 5105–5122

Semenov, A. (1985). Contribution to the theory of microphase layering in block-copolymer melts. *Sov. Phys. JETP* 61, 733–742

Zhulina, E., Adam, M., LaRue, I., Seiko, S., and Rubinstein, M. (2005). Diblock copolymer micelles in a dilute solution. *Macromolecules* 38, 5330–5351
